# Structure–Reactivity Relationships in Methylaluminoxane: Ionization Potentials Across Compositions and Morphologies

**DOI:** 10.1002/cphc.70583

**Published:** 2026-10-15

**Authors:** Xiaotong Mao, Wei Hu, Scott Collins, Mikko Linnolahti

**Affiliations:** ^1^ Department of Chemistry and Sustainable Technology University of Eastern Finland Joensuu Finland; ^2^ School of Chemistry and Chemical Engineering Qilu University of Technology (Shandong Academy of Sciences) Jinan Shandong China; ^3^ State Key Laboratory of Precision and Intelligent Chemistry University of Science and Technology of China Hefei Anhui China

**Keywords:** cage, ionization potentials, methylaluminoxane, reactivity, sheet

## Abstract

Methylaluminoxane (MAO) is the most widely used cocatalyst in metallocene‐catalyzed olefin polymerization, yet its structural basis for reactivity remains poorly understood. Here, we present a computational survey of ionization potentials across 118 MAO species with the general formula (AlOMe)_*n*_(AlMe_3_)_*m*_ (*n*  =  4–10), covering over 1100 aluminum sites classified by local coordination environment. The analysis is extended to experimentally relevant sizes through evaluation of sheet and cage models up to *n* = 18, including an ovalene‐type sheet model and crystallographically characterized 26,9 species. All energies are evaluated at the RI‐MP2 level. Ionization potentials become systematically more negative with increasing oligomer size, and sheet structures are more reactive than cages across the sizes studied. At experimentally relevant sizes, the reactive site landscape converges to two characteristic types: one for methide abstraction and one for dimethylaluminum cation cleavage, with the latter pathway becoming exceptionally favorable for the ovalene‐type sheet topology that matches the crystallographically confirmed MAO structure. The identification of specific sheet‐type species as the most reactive MAO components is consistent with the experimental observation that the catalytically active component of MAO is a minor species within the bulk material, providing a quantitative basis for understanding structure–reactivity relationships in this industrially important cocatalyst.

## Graphical abstract

Structure–reactivity relationships in methylaluminoxane (MAO) are quantified via RI‐MP2 ionization potential calculations. Ionization potentials become more negative with increasing oligomer size, and sheets are more reactive than cages. At experimentally relevant sizes, the superior reactivity of the ovalene‐type 16,7 sheet at its C‐type site explains why the catalytically active component of MAO is a minor species within the bulk material.

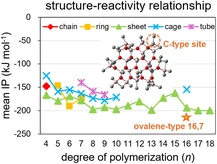

## 1 Introduction

Methylaluminoxane (MAO) is the most widely used cocatalyst in metallocene‐catalyzed olefin polymerization, yet its molecular‐level structure and the mechanism by which it activates the catalyst precursor remain incompletely understood [1, 2, 3, 4]. MAO is prepared by controlled hydrolysis of trimethylaluminum (TMA) [5, 6] and is generally described as a complex mixture of oligomeric aluminoxane species with the general formula (AlOMe)_*n*_(AlMe_3_)_*m*_, where *n* denotes the degree of polymerization and *m* the number of associated TMA units [7, 8, 9, 10]. The persistent structural ambiguity of MAO has motivated the development of well‐defined molecular alternatives, most notably an aluminum‐alkyl borate salt featuring a dinuclear Al cation, introduced as MAO’s “molecular cousin” [11], whose coordinately saturated aluminum centers exhibit the latent Lewis acidity thought to characterize the reactive sites of MAO itself and whose mechanism of action as a complete activator has recently been elucidated [12]. The success of such molecular analogs highlights how much of MAO’s own chemistry remains to be resolved at the level of individual aluminum sites. Over decades of study, a variety of experimental methods have provided important insights into the structure and reactivity of MAO [13, 14, 15, 16, 17, 18, 19, 20]. For example, bridging methyl groups present in MAO, detected by infrared spectroscopy, are crucial for metallocene catalyst activation as their replacement by bridging chloride groups leads to inactivation of MAO [21]. More recently, solid‐state ^27^Al NMR spectroscopy combined with theoretical calculations demonstrates that these bridging methyl sites are quite abundant in MAO (ca. 20 mol% of total), they can react with Lewis base donors (such as THF) or Cp_2_ZrMe_2_ to afford different reactive sites, and not all of these sites exhibit the same reactivity toward this complex [22]. Despite this significant progress, the structural and compositional heterogeneity of MAO has still made it highly challenging to characterize at the molecular level [23, 24]. Polymerization activity resides at the transition metal center of the metallocene cation, which is paired with an anion derived from MAO, but which MAO species furnish these anions and what structural features render them susceptible to ionization remain open questions.

Computational studies have played a central role in addressing this challenge [25, 26, 27, 28]. Systematic exploration of the (*n*,*m*) compositional space has revealed a rich structural landscape in which the dominant morphology evolves with oligomer size: small species form chains and rings, which give way to sheets and cages at intermediate sizes (*n* ≈ 4 and above), with tubular structures also emerging as a minor motif [8, 29, 30, 31]. Within this landscape, the thermodynamic balance between structural types is delicate. Sheets and cages are often close in electronic energy, but sheets tend to be favored in Gibbs free energy due to a vibrational entropy advantage, arising in part from weak and strained bonds at peripheral sites along the sheet edges that give rise to low‐frequency vibrations not found to the same extent in the more rigid cage topology [32, 33]. This entropy advantage is expected to diminish with increasing oligomer size, suggesting an eventual crossover to cage‐dominated thermodynamics at larger *n* [34, 35, 36], consistent with the expectation that the ultimate thermodynamic sink in these systems is alumina, a fully condensed and closed Al─O framework.

Experimental progress has accelerated in recent years. As shown in Figure 1, electrospray ionization mass spectrometry (ESI‐MS) studies have identified the [16,6]^−^ anion as the dominant species in MAO solutions [20, 37, 38], and we have recently shown computationally that this anion adopts a sheet structure analogous to an ovalene fragment of the infinite aluminoxane sheet [39]. This assignment gained strong support from the prior isolation and crystallographic characterization of a neutral (AlOMe)_26_(AlMe_3_)_9_ species, whose AlOMe core exhibits the same sheet topology and which is active in olefin polymerization [40]. The convergence of computational prediction and experimental confirmation thus points to sheet structures as a key reactive component of MAO [33, 41].

**FIGURE 1:**
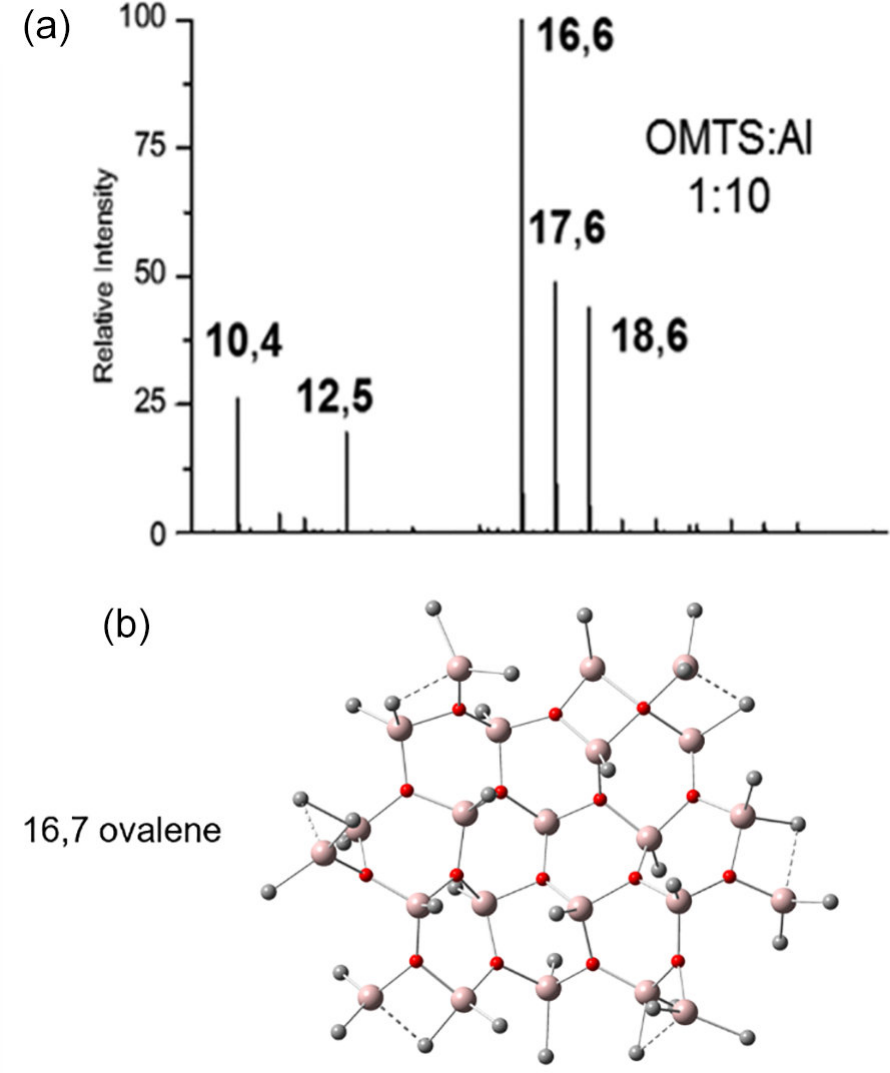
(a) Negative‐ion ESI‐MS spectrum of MAO recorded with OMTS as the donor (OMTS:Al = 1:10), in which the [16,6]^−^ anion dominates the distribution of (AlOMe)_*n*_(AlMe_3_)*_m_* species, together with the (b) 16,7 sheet structure identified as its precursor. Reproduced in part with permission from Ref. [37]. Copyright 2017, American Chemical Society.

However, it is well established that the catalytically active MAO species is present at low concentration within the bulk material [16, 42, 43]. This observation raises a fundamental question: what makes certain MAO structures more reactive than others? The activation of a metallocene precursor by MAO proceeds through ionization of the neutral MAO species to form an anionic cocatalyst, either by methide (Me⁻) abstraction from the metallocene by an aluminum center in MAO or by cleavage of a dimethylaluminum cation (Me_2_Al^+^) from the MAO framework [40, 44]. The feasibility of these ionization pathways can be probed computationally through ionization potentials (IPs), defined as the energy change associated with anion formation at each aluminum site relative to TMA and its anions as a reference. This metric, introduced in our earlier work [24], provides a site‐specific quantitative measure of the propensity of a given MAO structure to participate in catalyst activation. It should be noted that IPs measure only the thermodynamic feasibility of anion formation, a necessary but not sufficient condition for catalytic activation [2, 45]. Kinetic factors, including steric accessibility and substrate dependence, also play important roles during the activation process, but these are not captured by the definition of IP [46, 47].

Previous computational studies of MAO IPs have been limited in scope, covering only selected compositions and morphologies [24, 33, 48]. Moreover, it has become clear from recent benchmarking work that density functional theory (DFT) methods, including the widely used M06‐2X functional, do not provide reliable energetics for MAO systems, particularly for structures containing four‐coordinate oxygen sites that are characteristic of sheet and hybrid morphologies [49]. RI‐MP2 single‐point energies, validated against CCSD(T) reference calculations, are necessary for quantitatively meaningful results [50, 51, 52].

In this work, we present the first systematic survey of MAO IPs across a representative structural ensemble spanning *n* = 4–10, encompassing 118 distinct (AlOMe)_*n*_(AlMe_3_)_*m*_ species of all major morphological types (chains, rings, sheets, cages, and tubes) and covering over 1100 individual aluminum sites classified according to their local coordination environment. Treating all structures on an equal footing allows the reactivity advantage of sheets over cages to be established quantitatively, while site analysis shows that site occurrence does not correlate with reactivity. The analysis is extended to experimentally relevant sizes through the evaluation of sheet and cage models up to *n =* 18, together with the ovalene‐type 16,7 sheet model and crystallographically characterized 26,9 species, further revealing a simplified reactive site landscape at larger sizes. All IPs were evaluated at the RI‐MP2 level. The resulting reactivity landscape is analyzed as a function of composition, morphology, site type, and ionization mechanism, providing a comprehensive picture of structure–reactivity relationships in MAO and a quantitative basis for understanding why specific structural types dominate the reactive chemistry of this industrially important cocatalyst.

## 2 Computational Details

The structural ensemble investigated in this work comprises 118 distinct neutral MAO species with the general formula (AlOMe)_*n*_(AlMe_3_)_*m*_, spanning *n* = 4–10 and covering all major morphological types: chains, rings, sheets, cages, and tubes, as well as hybrid topologies featuring bridging AlO_4_ and AlO_5_ units. The structures were adopted from systematic, MP2‐level computational surveys of TMA hydrolysis products carried out in our group [50], reported previously up to *n* = 8 and extended here to *n* = 10 to reach the size domain where cage structures become energetically competitive with the sheets that dominate for *n* = 5‍–‍8. The structures were selected to provide representative coverage of the most stable species at each *n*,*m* composition. The complete set of structures, along with their morphological classifications and site labels, is provided in Figure S1.

Geometry optimizations of all neutral MAO species and their corresponding anions were performed using the M06‐2X density functional [53] with the TZVP basis set [54] in Gaussian 16 [55]. Although M06‐2X has known deficiencies for MAO energetics (Figure S6), our recent benchmarking study of 21 density functionals has shown that optimized geometries are relatively insensitive to the choice of functional [49]. Single‐point electronic energies were subsequently calculated at the RI‐MP2 [56]/def2‐TZVP [57] level of theory using ORCA 5.0.3 [58]. This computational protocol provides a practical balance between accuracy and the computational demands of treating over 2100 individual structures (118 neutral species and their anions generated at each unique aluminum site). All calculations were performed in the gas phase.

The local coordination environment of each aluminum site was classified according to a hierarchical scheme introduced in our earlier work for site types A through R (Figure 2) based on the number of Al─C bonds (with priority given to terminal over bridging methyl groups), the number of Al─O bonds, and the coordination number of the bound oxygen atoms [50]. The extension of the structural ensemble to *n*= 10 and the inclusion of cage and tube morphologies introduced additional site types (S through Z), which are cataloged in Figure S2.

**FIGURE 2:**
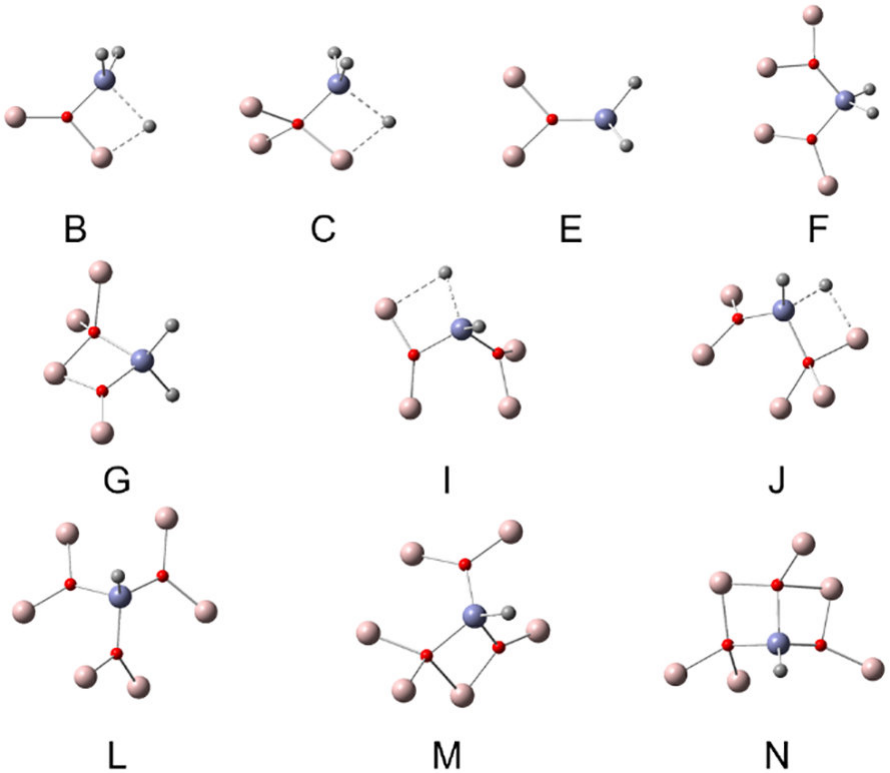
Classification of the most frequently encountered reactive Al sites (see Figure S2 for a complete list). The reactive site is highlighted in blue. The surrounding Al atoms are depicted in pink, O in red, and C in gray, with bridging C further identified by dashed lines. H atoms are omitted for clarity.

IPs were evaluated at each symmetry‐unique aluminum site through two pathways, both defined relative to TMA as a reference such that the ionization potential of TMA itself is zero by definition. The two pathways differ in the direction of fragment transfer. In the methide abstraction pathway, a Me^−^ group, which in catalyst activation originates from the metallocene precursor, is added to an aluminum center of a neutral *n*,*m* species to form the [*n*,*m*]^‒^ anion:

$$n\text{,}m\;+\;Me^{-}\;\to \;{\left[n\text{,}m\right]}^{-}$$

with the ionization potential defined as

$$\begin{array}{cc} \mathrm{IP}({\mathrm{Me}}^{-})\; & \text{= }E({\left[n\text{,}m\right]}^{-})\;\;-\;E(n\text{,}m)\;\;-\;\;[E(\mathrm{AlM}{e_{4}}^{-})\;\\ & \;-\;\text{½}E({\mathrm{Al}}_{2}{\mathrm{Me}}_{6})] \end{array}$$

In the Me_2_Al^+^ cleavage pathway, a Me_2_Al^+^ cation is removed from a TMA‐associated aluminum center through heterolytic scission of the Al─O framework:

$$n\text{,}m\;\to \;{\left[n\text{,}m-1\right]}^{-}\;+{\text{ Me}}_{2}{\text{Al}}^{+}$$

with

$$\begin{array}{ll} \mathrm{IP}({\mathrm{Me}}_{2}{\mathrm{Al}}^{+}) & \text{ =}\;\;E({\left[n\text{,}m-1\right]}^{-})\;-\;E(n\text{,}m)\;\\ & \;\;-\;[E(\mathrm{AlM}{e_{4}}^{-})-\;E({\mathrm{Al}}_{2}{\mathrm{Me}}_{6})] \end{array}$$

Here, IP(Me_2_Al^+^) corresponds to the quantity termed the anionization potential in our earlier work [24]. In both expressions, the bracketed reference terms represent the corresponding ionization energy of TMA, and the free species (Me^−^ and Me_2_Al^+^, respectively) cancel between the MAO and reference reactions. Negative IP values indicate that the MAO species ionizes more readily than TMA, with more negative values corresponding to higher reactivity. The Me_2_Al^+^ cleavage pathway is only accessible at sites bearing associated TMA units, whereas Me^−^ abstraction is not subject to this restriction and was therefore evaluated at every symmetry‐unique aluminum center.

## 3 Results and Discussion

### 3.1 Structural Ensemble and Site Classification

The 118 MAO species investigated here span a diverse structural landscape. At small oligomer sizes (*n* = 4), chains and rings coexist with the first sheet and cage structures, while at larger sizes (*n* = 8–10), sheets and cages dominate, supplemented by a small number of tubular structures. Across the full ensemble, sheets account for 46 structures, cages for 61, rings for 5, tubes for 5, and chains for 1. Representative structures are shown in Figure 3, and the complete catalog with labeled sites is provided in Figure S1.

**FIGURE 3:**
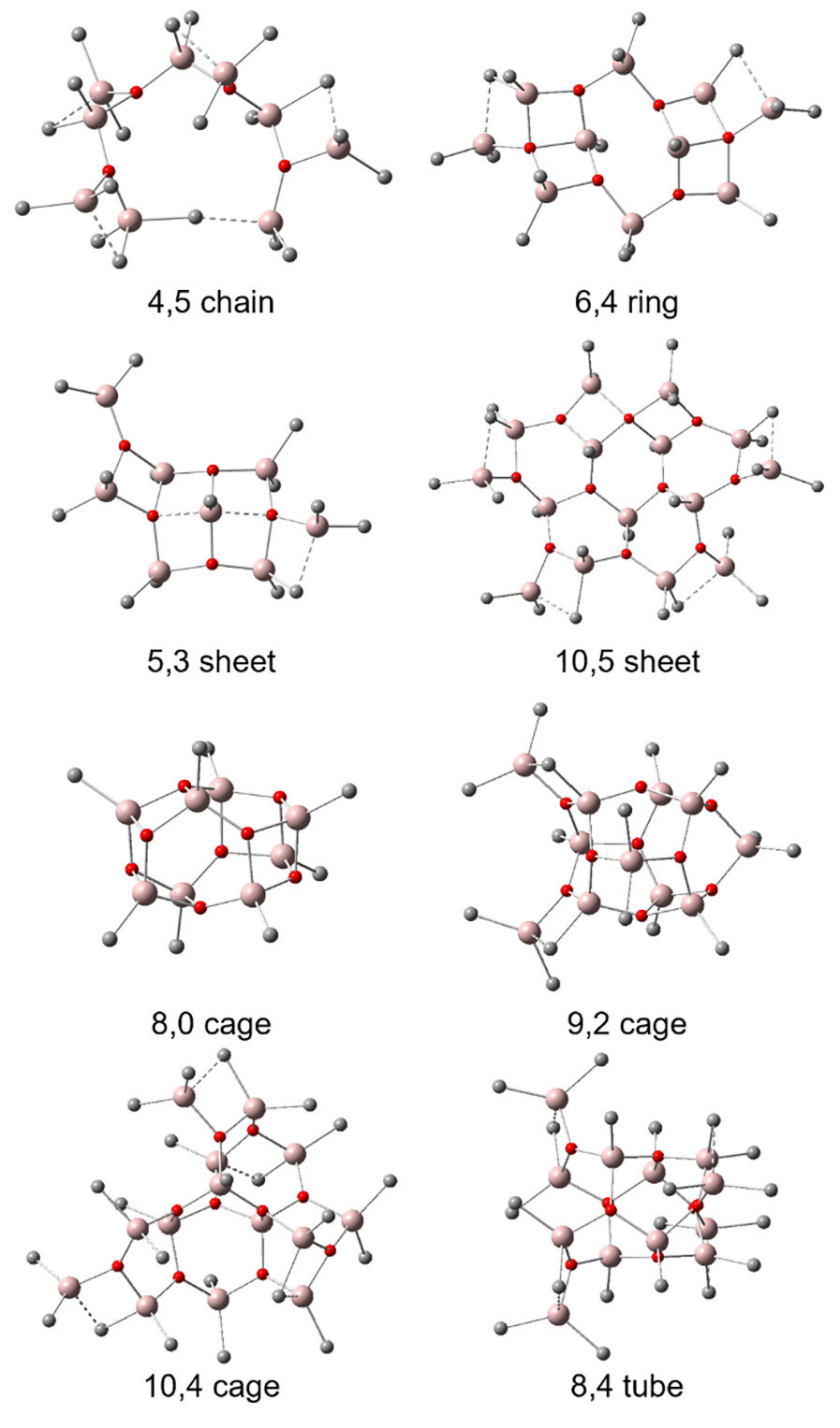
Representative optimized structures illustrating the major morphological types of MAO across the compositional range studied. Labels indicate the *n*,*m* composition and morphology; for compositions where multiple structural isomers are included, the complete catalog with individual structure identifiers is provided in Figure S1. Aluminum atoms are depicted in pink, oxygen in red, and carbon in gray. Hydrogen atoms are omitted for clarity.

A total of 26 distinct site types (A through Z) were identified based on the local coordination environment of each aluminum center (Figure S2). Sites I, L, F, and B are the most abundant across the ensemble, as plotted in the heatmap in Figure 4, reflecting the prevalence of four‐coordinate aluminum centers with one to three oxygen neighbors. The distribution of site types varies systematically with morphology: cage structures are dominated by I and L sites, whereas sheets exhibit a broader variety of site types, including G, J, M, and N alongside I and F. This difference in site diversity, as will be shown below, has direct consequences for reactivity.

**FIGURE 4:**
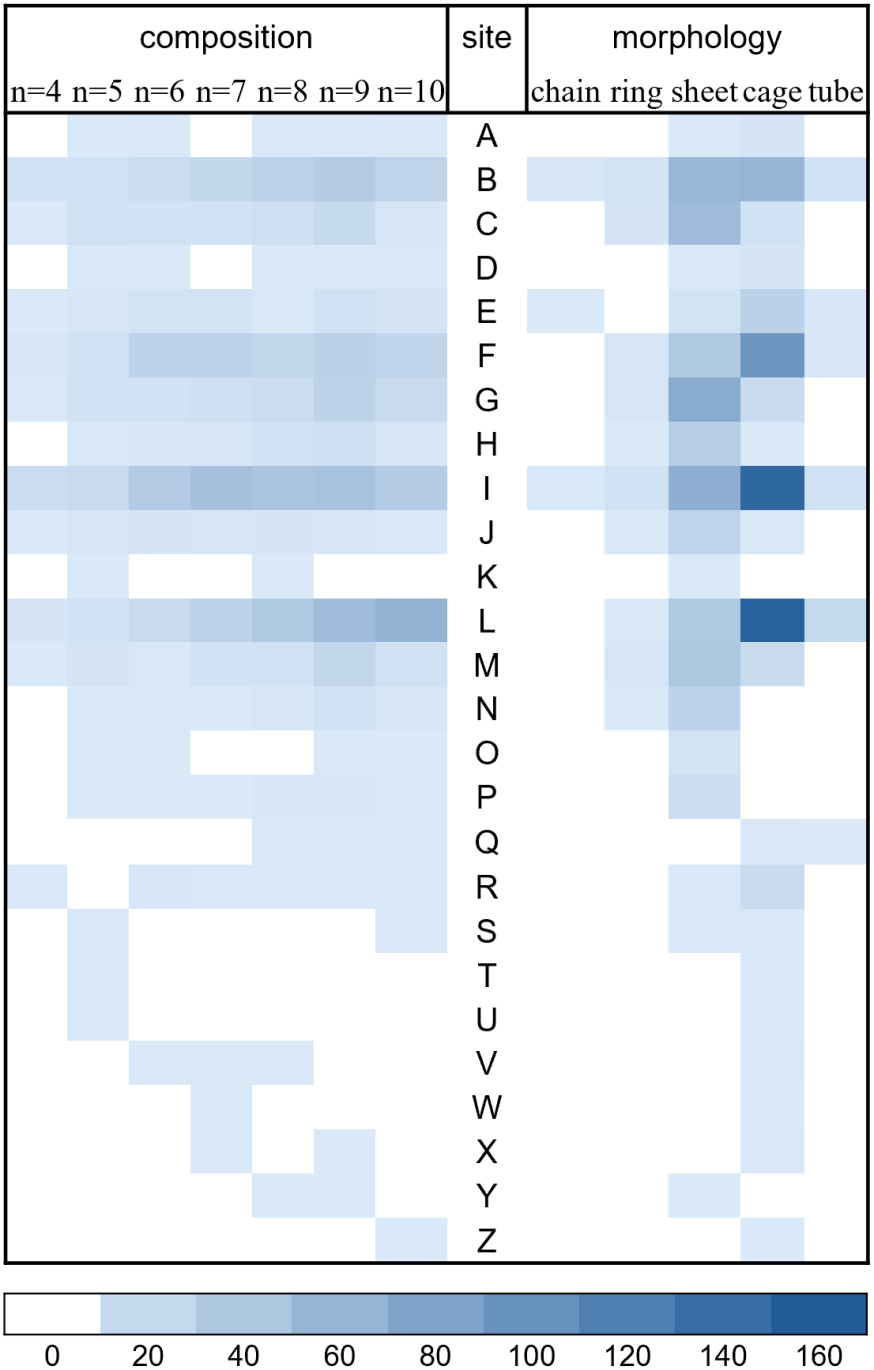
Heatmap of site occurrences with different compositions and morphologies.

### 3.2 Composition Dependence of IPs

The ionization potential of a given MAO species reflects the thermodynamic feasibility of anion formation at the most reactive aluminum site. To assess how molecular composition influences this reactivity, the lowest IP for each of the 118 neutral structures was examined as a function of the degree of polymerization (*n*) and the relative TMA content (*m*/*n* ratio), considering both ionization pathways.

The mean lowest IP per structure evolves from −143 kJ mol^–1^ at *n* = 4 to approximately −182 kJ mol^–1^ at *n* = 8, beyond which it levels off (the black line in Figure 5a). This trend is observed across all morphologies.

**FIGURE 5:**
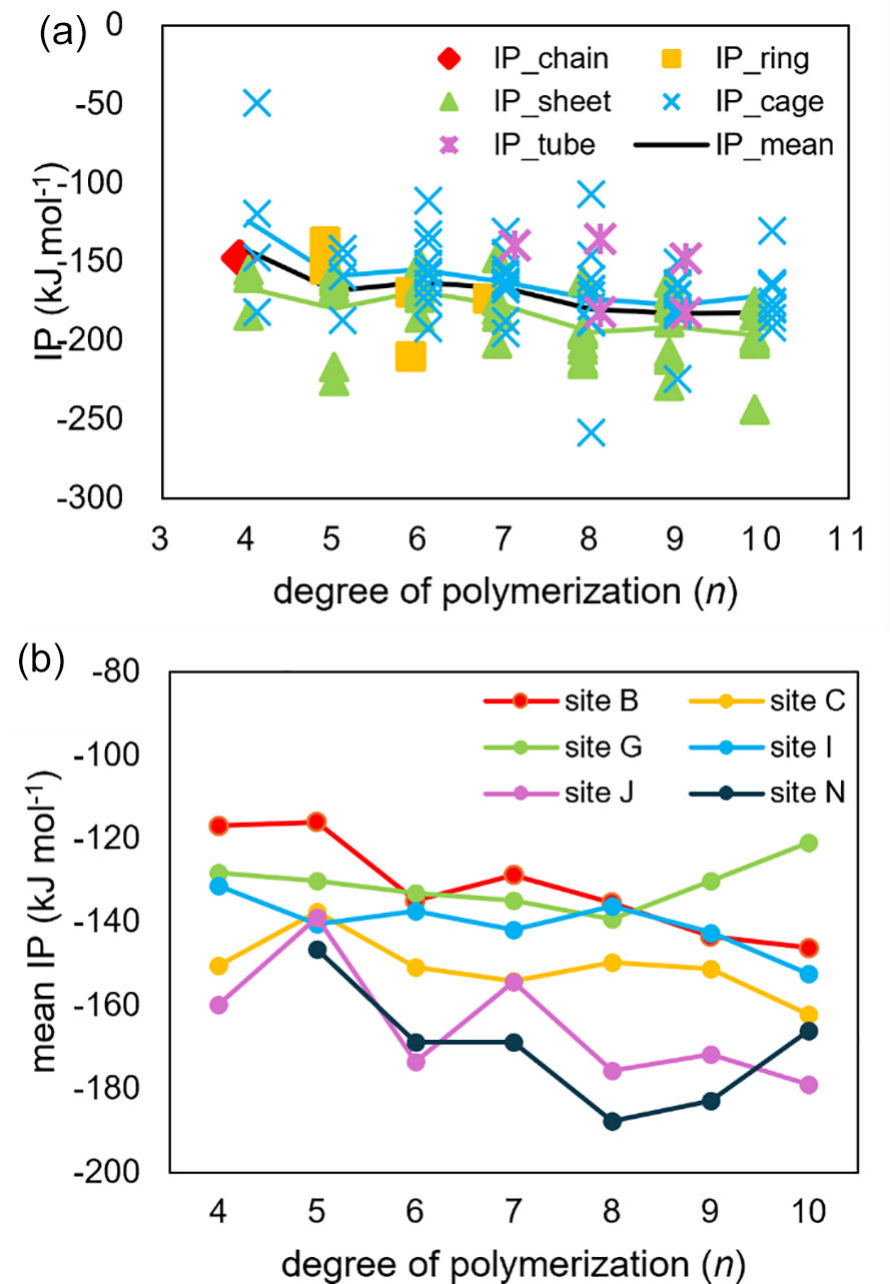
(a) Scatter plot of the lowest IP per structure (whichever pathway) as a function of the degree of polymerization (*n*), with data points colored by morphology. The trend lines (overall, sheet, and cage) of the mean lowest IP at each *n* are also included. (b) Line chart of the mean IP (Me^−^ abstraction) as a function of the degree of polymerization (*n*) for individual site types. The lines connecting the data points are included for visual guidance only.

The improvement in IPs with increasing *n* has two distinct origins. First, larger structures contain more aluminum sites and a greater diversity of site types, which increases the probability that at least one site achieves a strongly negative IP. The mean number of unique sites per structure grows from about 5 at *n* = 4 to 13 at *n* = 10. Second, for certain site types, the IP at a given site improves systematically with the framework size. For example, in Figure 5b, the mean IP(Me^−^) at B‐type sites decreases from −117 kJ ‍mol^−1^ at *n* = 4 to −146 kJ mol^−1^ at *n* = 10, and a similar trend is observed for I‐type sites. This indicates that the surrounding aluminoxane framework provides additional stabilization of the anionic product, most likely through the electrostatic and inductive effects of the polar Al─O network [19, 48]. The improvement of IPs at a given site type with increasing framework size is consistent with the ability of larger aluminoxane frameworks to accommodate the negative charge. It has been shown in the literature that large MAO oligomers are required for effective catalyst activation due to charge stabilization by the extended framework [13, 17, 24]. The present results support this picture but indicate that the size dependence of IP reflects both this electronic stabilization effect and the statistical effect of greater site diversity, with the relative importance of each contribution varying across site types.

The dependence on the *m*/*n* ratio is negligible (see Figure S3). Across the dataset, the lowest IP shows no systematic variation with relative TMA content, and the scatter at any given *m*/*n* spans essentially the full range of the data. Any apparent trend is governed by the TMA‐free n,0 structures, which are cage species composed almost entirely of core sites and are correspondingly unreactive; excluding them removes it. Relative TMA content therefore does not by itself govern reactivity in this dataset, which is instead determined by the morphology and local site structure.

### 3.3 Morphology Dependence of IPs

The structural morphology of MAO species has a pronounced effect on their IPs. Figure 6 compares the distributions of the lowest IP per structure for each morphological type. Sheets are the most reactive morphology, with a median lowest IP of −184 kJ mol^–1^ and a relatively compact distribution (interquartile range approximately −202 to −168 kJ mol^–1^). Cages are less reactive on average (median −165 kJ mol^–1^) but exhibit a wider distribution that extends to both less and more reactive extremes. Rings, tubes, and the single chain structure fall between these two, though the small sample sizes for these morphologies (five structures each for rings and tubes) limit the statistical significance of any comparison.

**FIGURE 6:**
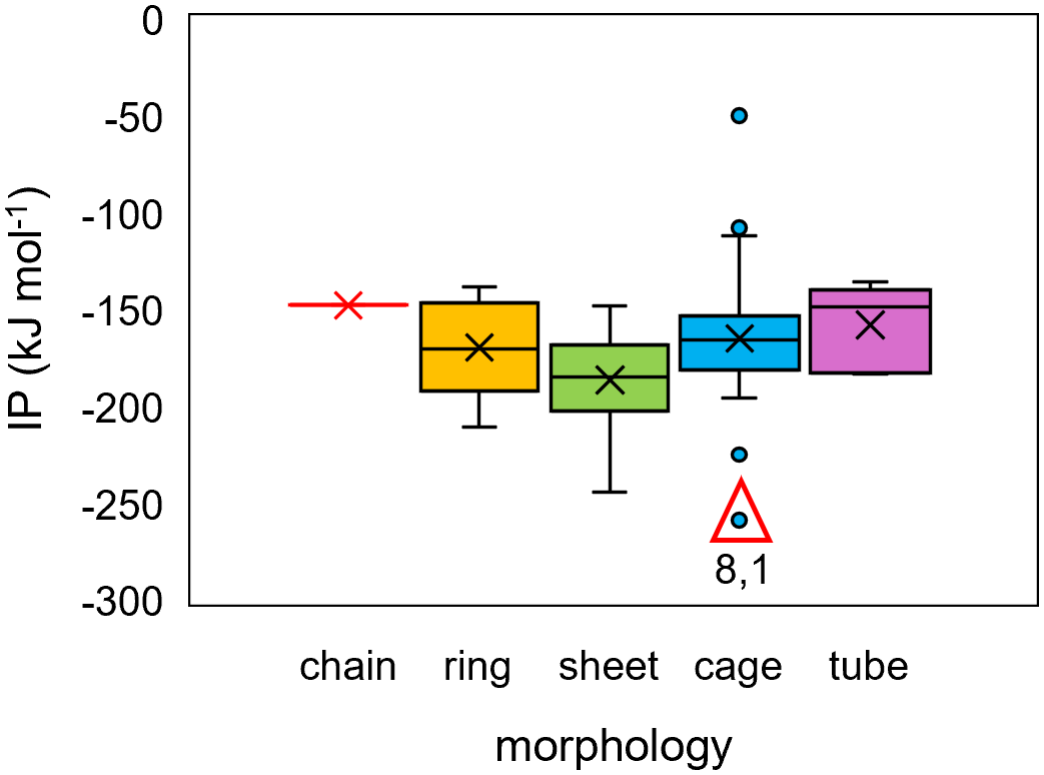
Box plot of the lowest IP per structure (whichever pathway) grouped by morphology. The 8,1 cage outlier (the structure with the lowest IP) is identifiable in the plot as a labeled point.

The reactivity advantage of sheets over cages is consistent across the full size range studied. As shown in Figure 5a, at each value of *n* from 4 to 10, the mean lowest IP for sheets is 14–42 kJ mol^–1^ more negative than for cages of comparable composition. This systematic difference points to a structural rather than compositional origin.

A key factor underlying this difference is the diversity of reactive site types accessible in each morphology. Among the 46 sheet structures, 12 distinct site types contribute as the most reactive site, with the three most frequent (N, I, and G) accounting for only 52% of structures. The open, two‐dimensional topology of sheets evidently supports a broad range of local coordination environments, many of which are favorable for ionization. In contrast, the 61 cage structures are dominated by just two site types: I and L account for 69% of all cases where a cage site gives the lowest IP. The closed, three‐dimensional cage topology constrains the available coordination environments, limiting the structural pathways to low IPs.

Among the cage structures, one outlier deserves a mention. The 8,1 cage achieves the single lowest IP in the entire dataset (−259 kJ ‍mol^–‍1^ at an L‐type site), far below that of any other structure. Inspection reveals that this structure contains two fused Al_2_O_2_ rings, a highly strained motif. Upon Me^−^ abstraction, the cage undergoes substantial reorganization, including the opening of the fused rings. The exceptionally low IP therefore reflects strain relief in the anionic product rather than intrinsic Lewis acidity at the reaction site and is not representative of the reactivity trends discussed here. In addition, the strained fused‐ring motif is a feature of small cages at low TMA content, while larger cage topologies, such as the 12,0 truncated octahedron, can close without such strained environments. The open questions for larger cages concern instead the influence of AlO_4_ units on the surrounding site reactivity. More generally, this example highlights the possibility that ionization‐induced structural reorganization may also affect the IP values of larger clusters. Whether this effect plays a widespread role in larger MAO species and whether it contributes systematically to activation behavior remains to be determined.

While sheets are more reactive than cages, the two morphologies are not equally represented across the thermodynamic landscape of MAO. Sheets dominate the most stable structures at intermediate sizes, but cages become increasingly competitive at larger *n*, particularly those incorporating AlO_4_ bridging units with four‐coordinate oxygen. Among the four 10,4 isomers in the present dataset, a cage featuring an AlO_4_ unit is favored in electronic energy over the sheet, cage, and mixed isomers of the same composition by 45–76 kJ mol^–1^. This comparison does not account for the higher vibrational entropies of sheets or their preference for greater TMA incorporation, both of which favor sheet structures in terms of Gibbs free energy at these sizes. Nevertheless, the electronic energy trend is consistent with earlier thermodynamic analyses, suggesting that cage structures with alumina‐like connectivity become increasingly competitive at larger oligomer sizes [34].

### 3.4 Site‐Type Dependence of IPs

The site‐type analysis provides the most granular view of MAO reactivity, connecting ionization behavior to the local coordination environment of individual aluminum centers. However, interpreting the site‐type data requires first recognizing that not all aluminum sites in MAO are genuinely reactive. The general requirement for reactivity is a deviation from the standard coordination pattern of the aluminoxane core: three‐coordinate oxygen, four‐coordinate aluminum, and terminal methyl groups. Sites that conform to this pattern, most notably L‐type sites that constitute the (AlOMe)_*n*_ backbone, are inherently poor Lewis acids and achieve low (strongly negative) IPs only under special circumstances.

L‐type sites are the most abundant in the dataset (212 occurrences) and illustrate this distinction clearly. The pure *n*,0 cages, which contain no associated TMA, are composed entirely of core aluminum sites. For *n* = 4–8, their IPs range from −49 to −132 kJ ‍mol^–1^, confirming their low intrinsic reactivity. However, larger *n*,0 cages can achieve substantially more negative IPs: the 9,0 cage reaches −171 kJ mol^–1^, and the 8,1 cage discussed above achieves −259 kJ mol^–1^. In both cases, the driving force is not intrinsic Lewis acidity at the aluminum center but rather the release of strain energy upon Me^−^ addition through the opening of fused Al_2_O_2_ rings (see Figure S4). This mechanism, termed latent Lewis acidity, is well established experimentally for structurally analogous *tert*‐butylaluminoxane cages [59, 60]. The distinction between intrinsic Lewis acidity at coordination‐deficient sites and latent Lewis acidity activated by structural reorganization is important for interpreting the trends that follow.

Similarly, F‐type sites represent AlMe_2_ moieties generated by the TMA association with the aluminoxane core. These sites share the standard coordination pattern of three‐coordinate oxygen, four‐coordinate aluminum, and terminal methyl groups and are correspondingly unreactive in most structural contexts (median IP = −65 kJ mol^–1^ across 144 occurrences). In the analysis below, both L and F are treated as core site types, and the focus is placed on aluminum centers whose coordination environments deviate from this pattern.

To focus the analysis on genuinely reactive sites, the six structures composed entirely of core‐type sites (*n*,0 cages for *n* = 4 and 6 through 10) were set aside, and the remaining 112 structures were analyzed according to which noncore site type provided the lowest IP. Figure 7a summarizes the statistical results and the corresponding site‐type images.

**FIGURE 7:**
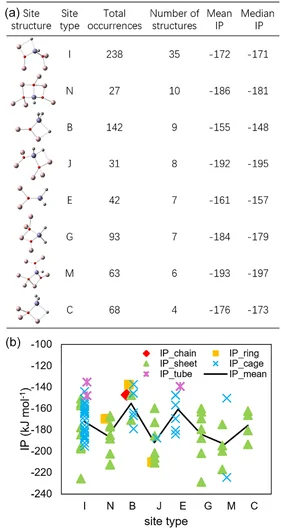
(a) Structures and statistics of noncore site types that give the lowest ionization potentials (kJ mol^–1^) in the *n* = 4–10 dataset. For each site type, the total number of occurrences in the dataset, the number of structures providing the lowest IP, as well as their mean IP and median IP are listed. (b) Scatter plot of the lowest IP and mean IP as a function of the noncore site types, colored by morphology.

Among the reactive site types, I remains the most frequent provider of the lowest IP per structure (35 of 112 structures), followed by N (10), B (9), J (8), and E (7). The dominance of I reflects its abundance across the structural ensemble (238 total occurrences) rather than exceptional intrinsic reactivity: only 15% of I‐type sites are the most reactive in their structure. The more revealing metric is the mean IP achieved when a given site type is the most reactive site. By this measure, sites M (−193 kJ mol^–1^), J (−192 kJ mol^–1^), N (−186 kJ mol^–1^), and G (−184 kJ mol^–1^) consistently outperform the more abundant I (−172 kJ mol^–1^) and B (−155 kJ mol^–1^). These four site types share a common structural feature: their local coordination environments deviate substantially from the standard core pattern, incorporating higher‐coordinate oxygen, bridging methyl groups, or combinations of both.

The distribution of these intrinsically reactive site types across morphologies provides a structural explanation for the reactivity advantage of sheets. As shown in Figure 7b, C‐type and G‐type sites occur exclusively in sheets (100% of all occurrences), as do N (90%) and J (75%). M‐type sites are also predominantly found in sheets (67%). These are precisely the site types that achieve the most negative IPs. In contrast, the most common reactive site type in cages is I (71% of all I occurrences are in cages), which, while frequently providing the lowest IP by virtue of its abundance, is intrinsically less reactive than the sheet‐characteristic sites M, J, N, and G. The open, two‐dimensional topology of sheets evidently supports a broader range of local coordination environments that deviate from the core pattern, and it is these deviations that confer the highest intrinsic reactivity.

### 3.5 Ionization Mechanism: Me^−^ Abstraction Versus Me_2_Al^+^ Cleavage

The two ionization pathways available to MAO differ fundamentally in their structural consequences. Me^−^ abstraction adds a methyl group to an aluminum center, converting *n*,*m* to the [*n*,*m*]^−^ anion without changing the TMA content, while Me_2_Al^+^ cleavage removes a TMA‐derived fragment, converting *n*,*m* to the [*n*,*m* − 1]^−^ anion [44]. Across the dataset, Me^−^ abstraction yields the lower IP in 113 of the 118 structures studied, with only five structures favoring Me_2_Al^+^. However, this apparent dominance must be interpreted with caution as the dataset composition introduces a systematic bias.

The structural ensemble, drawn from systematic hydrolysis studies, spans a range of *m* values at each *n*. At each oligomer size, the computationally most stable composition corresponds to a specific *m* value that increases with *n*, reflecting the growing number of peripheral sites available for TMA association. The dataset includes many structures with *m* values below this optimum as the hydrolysis surveys from which they were adopted systematically explore the full compositional range. At low *m*, the neutral MAO has fewer TMA‐derived sites than the computationally most stable structure at that size. Me^−^ abstraction, which does not deplete the TMA, is naturally favored under these conditions, while Me_2_Al^+^ cleavage, which removes a TMA unit, incurs an additional energetic penalty by driving the composition further from the optimum. This compositional bias in the dataset means that the apparent dominance of Me^−^ abstraction (113 of 118 structures) partly reflects the overrepresentation of low‐*m* compositions rather than an intrinsic preference of MAO for this pathway.

Consistent with this reasoning, within the low‐molecular‐weight ensemble studied here, the pathway preference depends on the *m*/*n* ratio. At *m*/*n* < 0.7, Me^−^ abstraction is preferred in all but one of 98 structures, whereas at *m*/*n* ≥ 0.7, Me_2_Al^+^ cleavage becomes competitive, accounting for 4 of 20 structures (20%). The five structures favoring Me_2_Al^+^ are four sheets (6,5, 7,5, 8,6, and 10,6) and one cage (6,5), all with *m*/*n* ratios between 0.60 and 0.83, and the energy differences between the two pathways are modest (1–9 kJ mol^–1^). The *m*/*n* = 0.7 boundary carries no significance beyond this dataset and simply marks where, among the small oligomers studied, TMA‐rich compositions begin to support competitive Me_2_Al^+^ cleavage. Whether a comparable balance holds for MAO species in the experimental size range is examined in the following section through the extension of the analysis to larger sheet structures.

### 3.6 IPs in the Experimental Size Range

The analysis above is limited to *n* = 4–10. While this range allows systematic exploration of composition and morphology effects, it falls below the molecular weight and above the composition range established for MAO by experiment. Cryoscopic, scattering, and diffusion measurements place the average molar mass of commercial MAO in the range of roughly 1100–1800 g mol^−1^, corresponding to aluminoxane frameworks substantially larger than those in the systematic dataset [17], while the average composition of MAO corresponds to *m/n* values between 0.25 and 0.42 [61]. To assess whether the trends identified above extrapolate to this experimentally relevant size and composition range, the analysis was extended to larger sheet structures using models reported in our previous work [33, 62]. Specifically, RI‐MP2/def2‐TZVP single‐point energies were computed for the 4‐coordinate (4‐C) sheet structures with *n* = 11 to 18 and their most reactive anions, as originally reported at the M06‐2X level, along with the isomeric cage models for compositions 16,6 and 16,7. In addition, the ovalene‐type 16,7 sheet and its [16,6]^−^ anion [39], recently identified as the structural model for the dominant MAO anion observed by ESI‐MS and closely related to the crystallographically characterized 26,9 species [40], were included as reference points. Representative structures are shown in Figure 8, and the extended catalog with labeled sites is provided in Figure S5. In all cases, the M06‐2X/TZVP‐optimized geometries from the original publications were retained, and only the single‐point energies were recomputed at the RI‐MP2 level, consistent with the computational protocol used for the *n* = 4–10 dataset. The results are summarized in Table 1.

**FIGURE 8:**
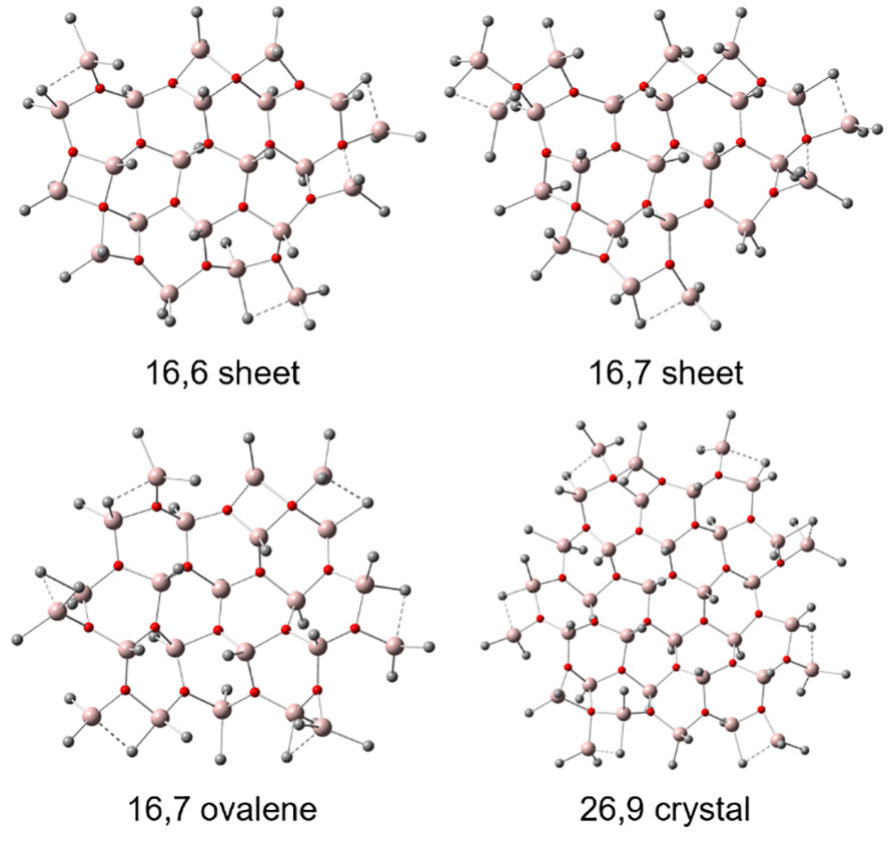
Representative optimized structures within the experimental size range studied. Labels indicate the *n*,*m* composition and morphology; complete catalog of extended MAO structures (*n* = 11–18) is provided in Figure S5. Aluminum atoms are depicted in pink, oxygen in red, and carbon in gray. Hydrogen atoms are omitted for clarity.

**TABLE 1:** Ionization potentials (kJ mol^–1^) at the RI‐MP2/def2‐TZVP level for sheet and cage structures in the experimental size range (*n* = 11–18). For each structure and pathway, the most reactive site type and its IP are listed. The ovalene 16,7 and crystal structure 26,9 models are included as reference points from previous work [39, 40].

*n*,*m*	Morphology	Me_2_Al^+^ cleavage		Me^‒^ abstraction	
		Site type	IP	Site type	IP
11,5	4‐C sheet	C	−164	G	−203
11,6	4‐C sheet	B	−143	M	−175
12,5	4‐C sheet	C	−160	G	−192
12,6	4‐C sheet	C	−146	B	−168
13,5	4‐C sheet	H	−153	G	−210
13,6	4‐C sheet	C	−157	G	−187
14,6	4‐C sheet	C	−141	G	−184
14,7	4‐C sheet	C	−160	—	—
15,6	4‐C sheet	C	−152	G	−181
15,7	4‐C sheet	C	−143	—	—
16,6	4‐C sheet	C	−165	G	−199
16,6	cage	A	−158	F	−58
16,7	4‐C sheet	C	−160	G	−188
16,7	cage	A	−149	B	−120
17,6	4‐C sheet	C	−164	G	−200
17,7	4‐C sheet	C	−165	G	−200
18,6	4‐C sheet	C	−164	G	−199
18,7	4‐C sheet	C	−167	G	−201
16,7	ovalene	C	−215	—	—
26,9	crystal	C	−217	—	—

The results for the 4‐C sheets confirm that the trends identified in the *n* = 4–10 dataset extend to experimentally relevant sizes while also revealing a significant simplification of the reactive site landscape at larger oligomer sizes. For the Me^−^ pathway, G‐type sites provide the lowest IP in nearly all 4‐C sheet structures for *n* ≥ 13, with values plateauing around −200 kJ mol^–1^ for *n* ≥ 16. This represents a further decrease of approximately 20 kJ mol^−1^ relative to the mean of −182 kJ mol^–1^ observed for sheets at *n* = 8–10, indicating that the electronic stabilization of the anionic product by the extended framework continues beyond the range of the systematic dataset. For the Me_2_Al^+^ pathway, C‐type sites emerge as the dominant reactive environment, with IPs converging to −160 to −167 kJ mol^−1^ at larger sizes. This convergence to just two characteristic reactive site types (G for Me^−^ and C for Me_2_Al^+^) contrasts with the broader diversity of reactive sites observed in the *n* = 4–10 dataset, suggesting that much of the site‐type diversity identified at smaller sizes reflects the limited extent of the sheet topology in those oligomers rather than features of experimentally relevant MAO structures.

The comparison between sheets and cages shows sheets to be the more reactive morphology by either pathway within the static picture considered here. The 4‐C sheet 16,6 achieves IPs of −165 (Me_2_Al^+^, site C) and −199 (Me^−^, site G), while the isomeric cage reaches only −158 (Me_2_Al^+^, site A) and −58 (Me^−^, site F). At composition 16,7, the comparison is similar: the 4‐C sheet gives −160 and −188, compared to −149 and −120 for the cage. The reactivity of sheets is straightforwardly understood in terms of the well‐defined edge sites (C and G types) that are a characteristic feature of the open‐sheet topology. The situation for cages is less clear. The closed cage topology does not offer equivalent edge environments, and the ionization behavior depends strongly on the specific cage structure, with the most reactive cage site type and its IP differing substantially even between the two compositions studied here. Both the 16,6 and 16,7 cages are most reactive toward Me_2_Al^+^ cleavage. This pathway reflects the thermodynamic feasibility of anion formation only, and whether cages of this type react in this manner toward weak donors such as metallocenes is a separate question that lies beyond the scope of the present analysis. Cage reactivity may also be influenced by dynamic processes that the static structures considered here do not capture, such as the reversible dissociation of coordinated AlMe_3_ [62], which can generate E‐type OAlMe_2_ reactive sites absent from the parent structure. The present comparison is limited to the two cage models previously reported at this size, and a systematic exploration of cage reactivity in the experimental size range would be needed to draw definitive conclusions about the relative activation capability of sheets and cages in MAO.

The most striking result concerns the ovalene‐type 16,7 sheet. Across the 4‐C sheet series, Me^−^ abstraction at G‐type sites (converging to approximately −200 kJ mol^–1^) is consistently more favorable than Me_2_Al^+^ cleavage at C‐type sites (−160 to −167 kJ mol^–1^). The ovalene topology reverses this relationship. Me_2_Al^+^ cleavage from the C‐type site of the ovalene 16,7 achieves an IP of −215 kJ mol^–1^, which is 50 kJ mol^–1^ more negative than the best Me_2_Al^+^ value for any 4‐C sheet and 15 kJ mol^–1^ more negative than the best Me^−^ value at any G‐type site for *n* = 16 – 18. The 26,9 crystal structure model yields a nearly identical value of −217 kJ mol^–1^ at the same site type. The ovalene model shares the same fundamental sheet topology as the crystallographically characterized 26,9 structure, and this topology gives rise to edge sites that are especially favorable for Me_2_Al^+^ cleavage. The effect is sensitive to the detailed edge structure, with the most stable anion isomer (O3) giving an IP of −215 kJ mol^−1^ and the alternative isomer (O4a) giving −186 kJ mol^−1^ [39]. The consequences of these edge differences for ion pair reactivity and olefin insertion have been examined separately [63]. The 4‐C sheets located by systematic hydrolysis, although closely related in connectivity, do not reproduce this edge arrangement and show a correspondingly less favorable Me_2_Al^+^ cleavage.

These results provide a direct connection between the computational reactivity landscape and the dominant experimental observable in MAO chemistry. The [16,6]^−^ anion that dominates ESI‐MS spectra is best understood as arising from Me_2_Al^+^ cleavage from the ovalene‐type 16,7 sheet at a C‐type site, a pathway that is by a wide margin the most favorable ionization process identified in this study. While only a limited number of structural topologies have been explored at this size, the ovalene model is distinguished by its close structural relationship to the crystallographically characterized 26,9 species, lending confidence that it represents a physically relevant MAO component. A systematic exploration of alternative sheet topologies in the experimental size range remains an open challenge.

## 4 Conclusion

A systematic survey of IPs across 118 MAO species spanning (AlOMe)_*n*_(AlMe_3_)_*m*_ compositions from *n* = 4–10 has been presented, covering over 1100 individual aluminum sites evaluated at the RI‐MP2 level of theory. The resulting reactivity landscape, extended to experimentally relevant sizes through analysis of sheet and cage models up to *n* = 18 and the crystallographically characterized 26,9 species, reveals how composition, morphology, local site structure, and sheet topology jointly determine the propensity of MAO to form anionic cocatalyst species.

IPs become systematically more negative with increasing oligomer size, with the mean lowest IP per structure evolving from −143 kJ mol^–1^ at *n* = 4 to approximately −182 kJ mol^–1^ at *n* = 8–10 for the systematic dataset and decreasing further to approximately −200 kJ mol^–1^ for sheet structures at *n* = 16–18. This improvement reflects both greater site diversity and electronic stabilization of the anionic product by the extended aluminoxane framework.

Within the static picture captured by the IPs computed here, sheet structures are consistently more reactive than cages across all sizes studied, as seen in the direct sheet‐cage comparison at the composition (*n* = 16) of the dominant MAO anion observed by ESI‐MS. This comparison does not account for the dynamic behavior of cage‐coordinated AlMe_3_, which can dissociate reversibly to expose reactive sites absent from the static structure, whereas sheets possess only structural AlMe_3_. A full account of the relative reactivity of the two morphologies would need to incorporate these dynamic effects. In the systematic dataset (*n* = 4–10), a range of site types contribute to sheet reactivity. At larger sizes, the reactive site landscape simplifies to two characteristic types: G for Me^−^ abstraction and C for Me_2_Al^+^ cleavage. The site diversity observed at smaller sizes thus partly reflects the limited extent of the sheet topology in those oligomers. For cages, the present comparison is restricted to two previously reported models at *n* = 16, and a more systematic exploration of cage reactivity in the experimental size range remains an open task.

The analysis of the ionization mechanism reveals that within the systematic dataset, the apparent dominance of Me^−^ over Me_2_Al^+^ cleavage is partly an artifact of the overrepresentation of low‐*m* compositions. At *m*/*n* ratios closer to the computationally preferred values, the two pathways become competitive. The extension to larger sheets demonstrates that the preferred pathway depends critically on the sheet topology. For the 4‐C sheet series, Me^−^ abstraction at G‐type sites (approximately −200 kJ mol^–1‍^‍) is consistently more favorable than Me_2_Al^+^ cleavage at C‐type sites (−160 to −167 kJ mol^–1^). However, the ovalene‐type 16,7 sheet achieves −215 kJ mol^–1^ for Me_2_Al^+^ cleavage at its C‐type site, a value confirmed by the 26,9 crystal structure model at −217 kJ mol^–1^. This is by far the most favorable ionization identified in this study and rationalizes why the [16,6]^−^ anion dominates the ESI‐MS spectrum: it arises from Me_2_Al^+^ cleavage from a structurally specific sheet precursor whose topology matches the crystallographically confirmed MAO component.

The finding that sheet structures, and in particular the ovalene‐type topology, are the most reactive MAO components identified in this study is consistent with the experimental observation that the catalytically active component of MAO is a minor species within the bulk material. The reactive sheet species represent only a fraction of the complex mixture of oligomers that constitutes MAO, yet they are the ones that produce the anions detected by ESI‐MS and that participate most readily in catalyst activation.

Looking ahead, the present study operates within the framework of gas‐phase electronic energies. Incorporation of Gibbs free energies, solvent effects, and aggregation phenomena represents the natural next steps. The sheet advantage holds within the static picture considered here, and the limited cage sampling is the reason a systematic exploration would be required. The dataset compiled in this work, comprising over 1100 site‐specific IPs with consistent site classifications across a diverse structural ensemble, provides a foundation for machine learning approaches aimed at predicting the reactivity of MAO species, including cage structures, beyond the size range accessible to direct quantum chemical calculation.

## Funding

This study was supported by the China Scholarship Council (202207960014) and Research Council of Finland (357509).

## Conflicts of Interest

The authors declare no conflicts of interest.

## Supporting information

Supplementary Material

## Data Availability

The data that support the findings of this study are available in the Supporting Information of this article.
