# Supplementary material for: Structure–Reactivity Relationships in Methylaluminoxane: Ionization Potentials Across Compositions and Morphologies
Source: Chemphyschem. 2026 Oct 15;27(20):e70583. doi: 10.1002/cphc.70583 (PMC13671876; doi:10.1002/cphc.70583)
Supplement: Supplementary file 1 [file CPHC-27-e70583-s001.pdf]

Supporting Information for

Structure-Reactivity Relationships in Methylaluminoxane: Ionization Potentials Across Compositions and Morphologies

Xiaotong Mao, Wei Hu, Scott Collins, Mikko Linnolahti*

Supporting Information 1
Figure S-1 – Complete MAO structures list (*n* = 4-10) with labeled reactive sites. 4
Figure S-2 – Site classifications. 4
Figure S-3 – Scatter plot of the lowest IP as a function of the relative TMA content (*m*/*n* ratio). 5
Figure S-4 – Structural comparison of 9,0 cage before and after Me^{-} abstraction. 5
Figure S-5 – Extended MAO structures list (*n* = 11-18) with labeled reactive sites. 6
Figure S-6 – Box plots of the IP deviations of M06-2X from RI-MP2 by site classification. 6
Table S-1 – Gas-phase electronic energies (a.u.) at the RI-MP2/def2-TZVP level of theory for species (*n* = 4 to 10) reported in Figure 3(a). Coordinates of neutrals and anions appear in Table S-1.xyz and Table S-2.xyz, respectively. 6
Table S-2 – Gas-phase electronic energies (a.u.) at the RI-MP2/def2-TZVP level of theory for species reported in Table 2. Coordinates of neutrals and anions appear in Table S-3.xyz 9

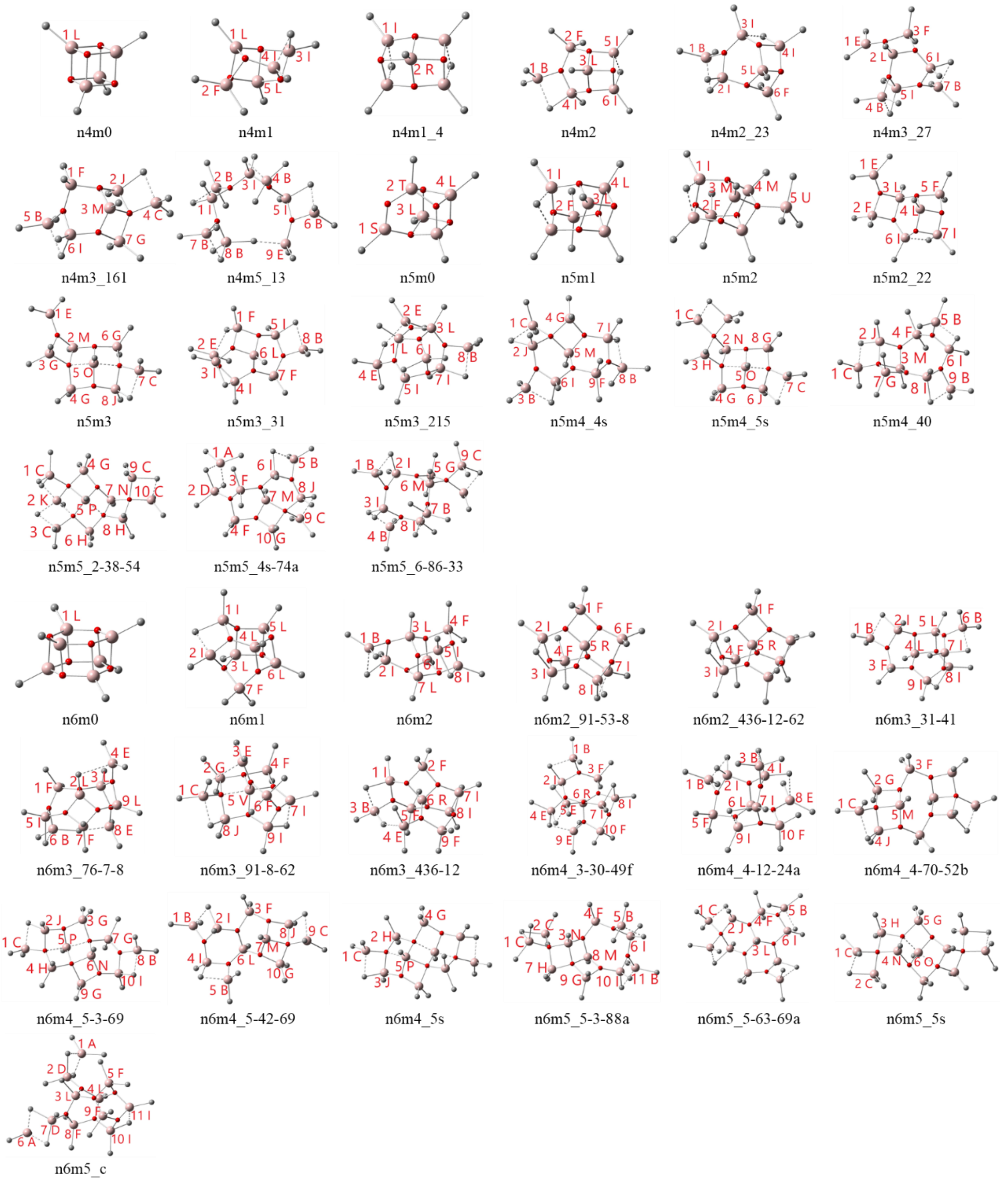

n4m0 n4m1 n4m1_4 n4m2 n4m2_23 n4m3_27

n4m3_161 n4m5_13 n5m0 n5m1 n5m2 n5m2_22

n5m3 n5m3_31 n5m3_215 n5m4_4s n5m4_5s n5m4_40

n5m5_2-38-54 n5m5_4s-74a n5m5_6-86-33

n6m0 n6m1 n6m2 n6m2_91-53-8 n6m2_436-12-62 n6m3_31-41

n6m3_76-7-8 n6m3_91-8-62 n6m3_436-12 n6m4_3-30-49f n6m4_4-12-24a n6m4_4-70-52b

n6m4_5-3-69 n6m4_5-42-69 n6m4_5s n6m5_5-3-88a n6m5_5-63-69a n6m5_5s

n6m5_c

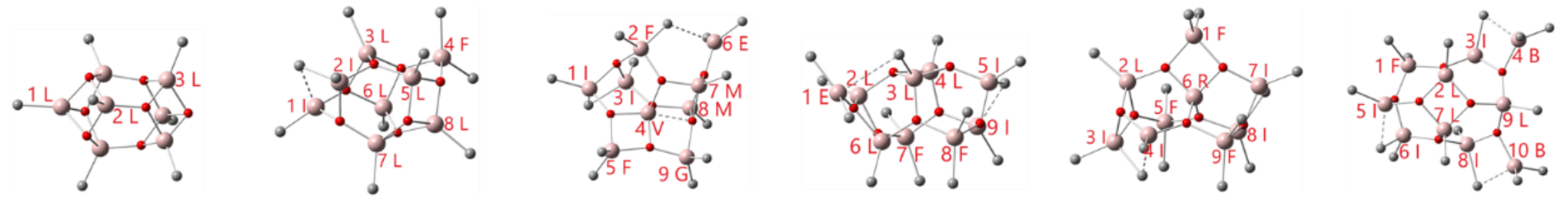

n7m0_c3v n7m1 n7m2_2-17 n7m2_3-35-50 n7m2_3-72-30 n7m3_3-26-43b

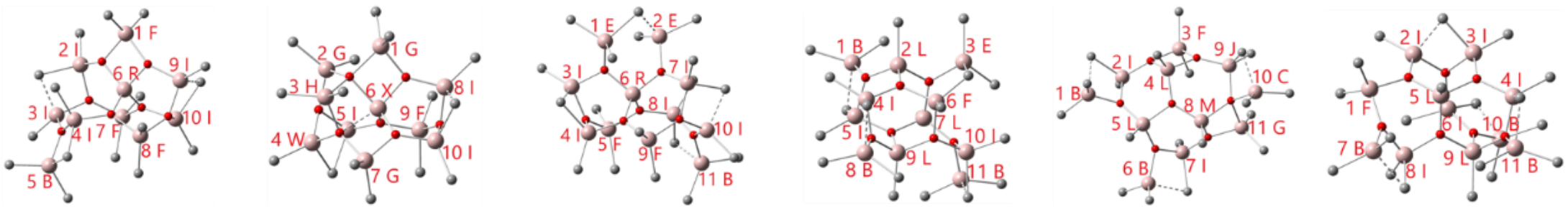

n7m3_3-62 n7m3_3-62-48a n7m4_3-62c n7m4_4-22-49b n7m4_4-34-63a n7m4_4-67-43a

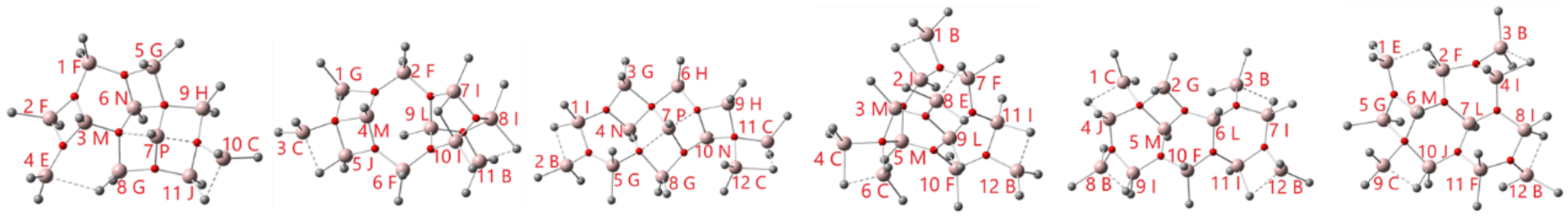

n7m4_5-25-90 n7m4_5-63-68 n7m5_5-21-3a n7m5_5-76-3a n7m5_5-82 n7m5_5-89

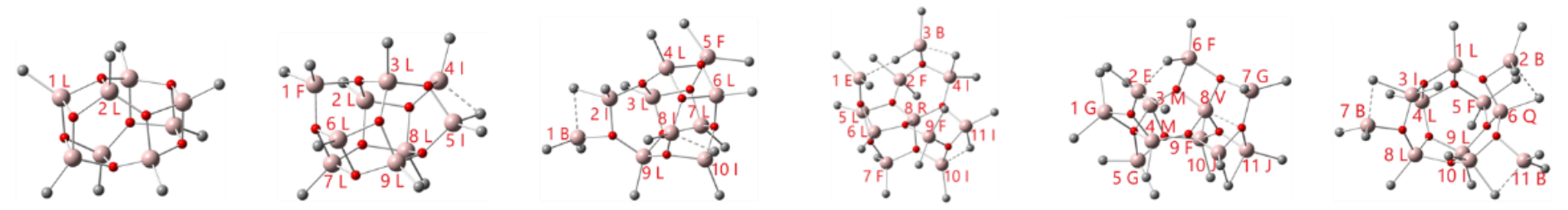

n8m0 n8m1_2-45-57 n8m2_2-61-37a n8m3_3-7 n8m3_3-21-7 n8m3_3-74-54a

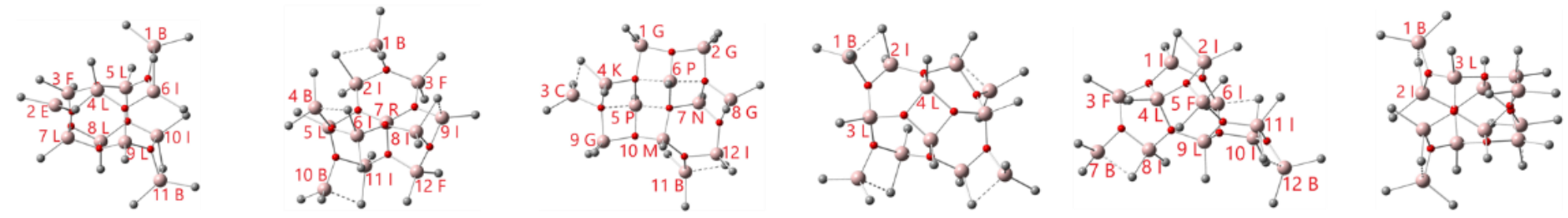

n8m3_4-91-13 n8m4_3-26c n8m4_4-41 n8m4_4-63-35a n8m4_4-91-24a n8m4_tube

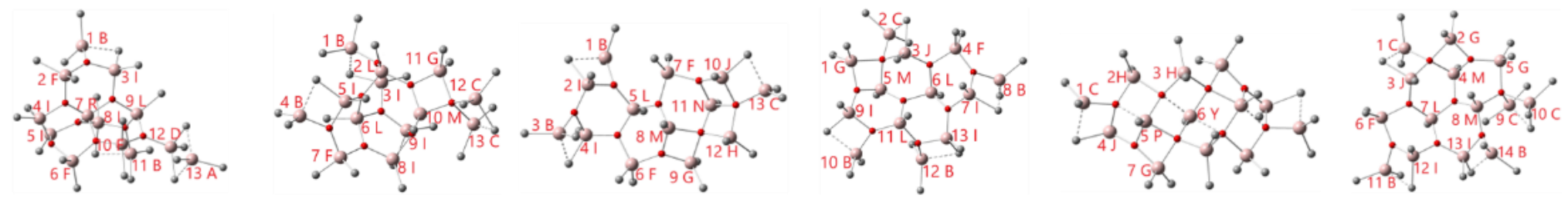

n8m5_4-69b n8m5_4-83a n8m5_5-12-53a n8m5_5-68 n8m5_5-87 n8m6_5-104-21c

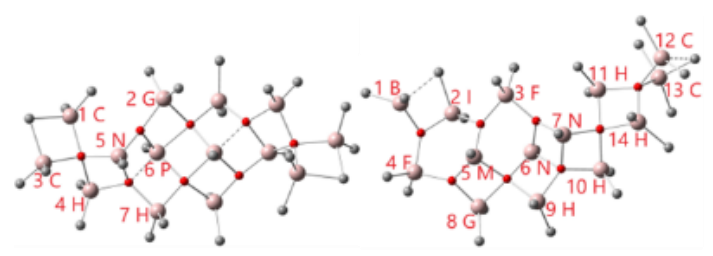

n8m6_5s n8m6_sc

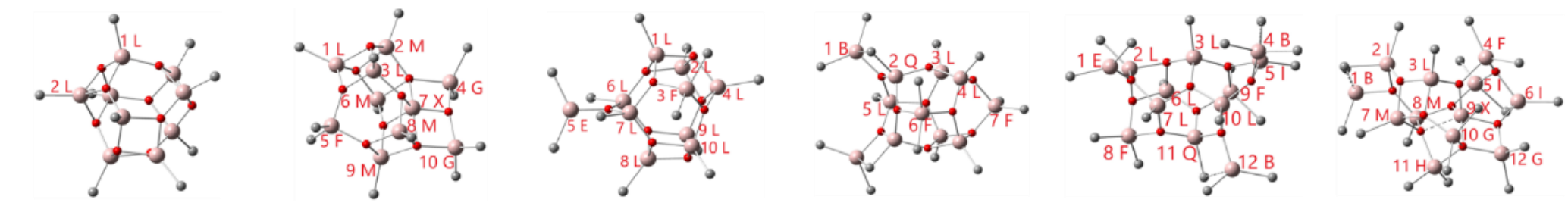

n9m0 n9m1_1-13 n9m1_1-54-36a n9m2_2-36-55a n9m3_1-20h n9m3_3-25-9a

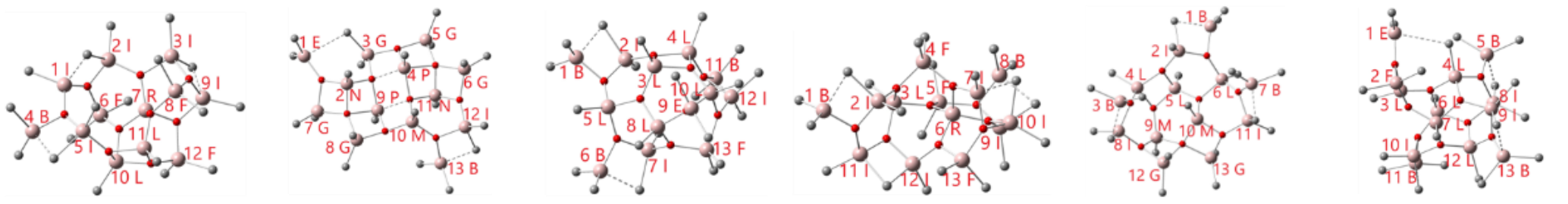

n9m3_3-74-39a n9m4_4-68 n9m4_4-93-65a n9m4_4-97-83a n9m4_4s n9m4_tube

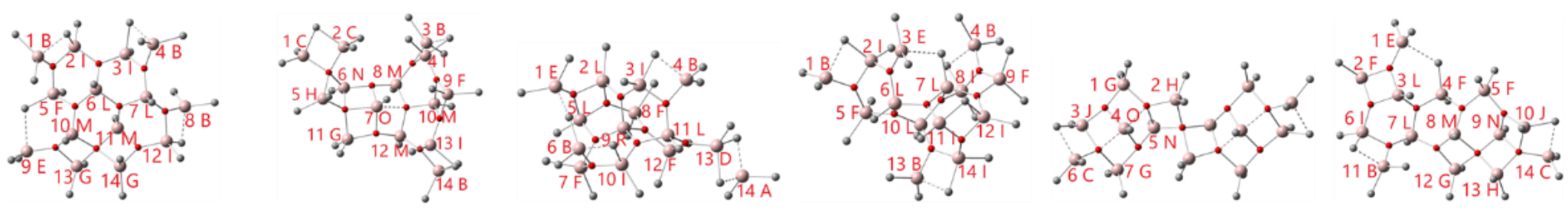

n9m5_5-5-3 n9m5_5-13 n9m5_5-54 n9m5_5-93 n9m5_5s n9m5_5-sc-106

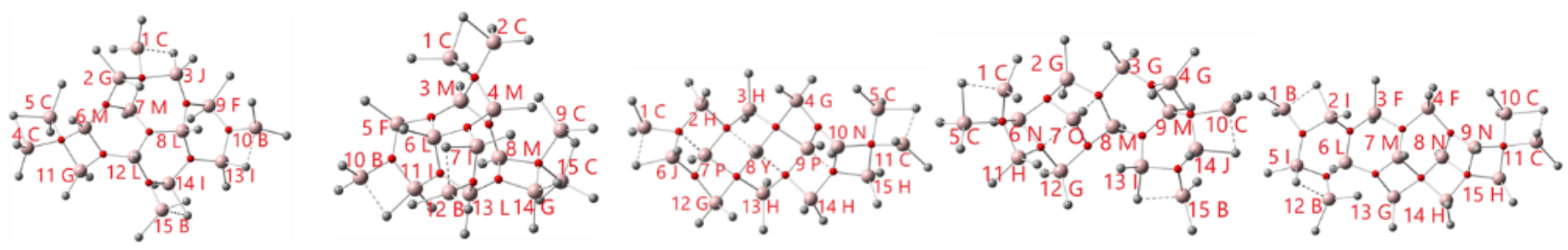

n9m6_5-5-100a n9m6_5-44a n9m6_5s n9m6_6-110 n9m6_sc

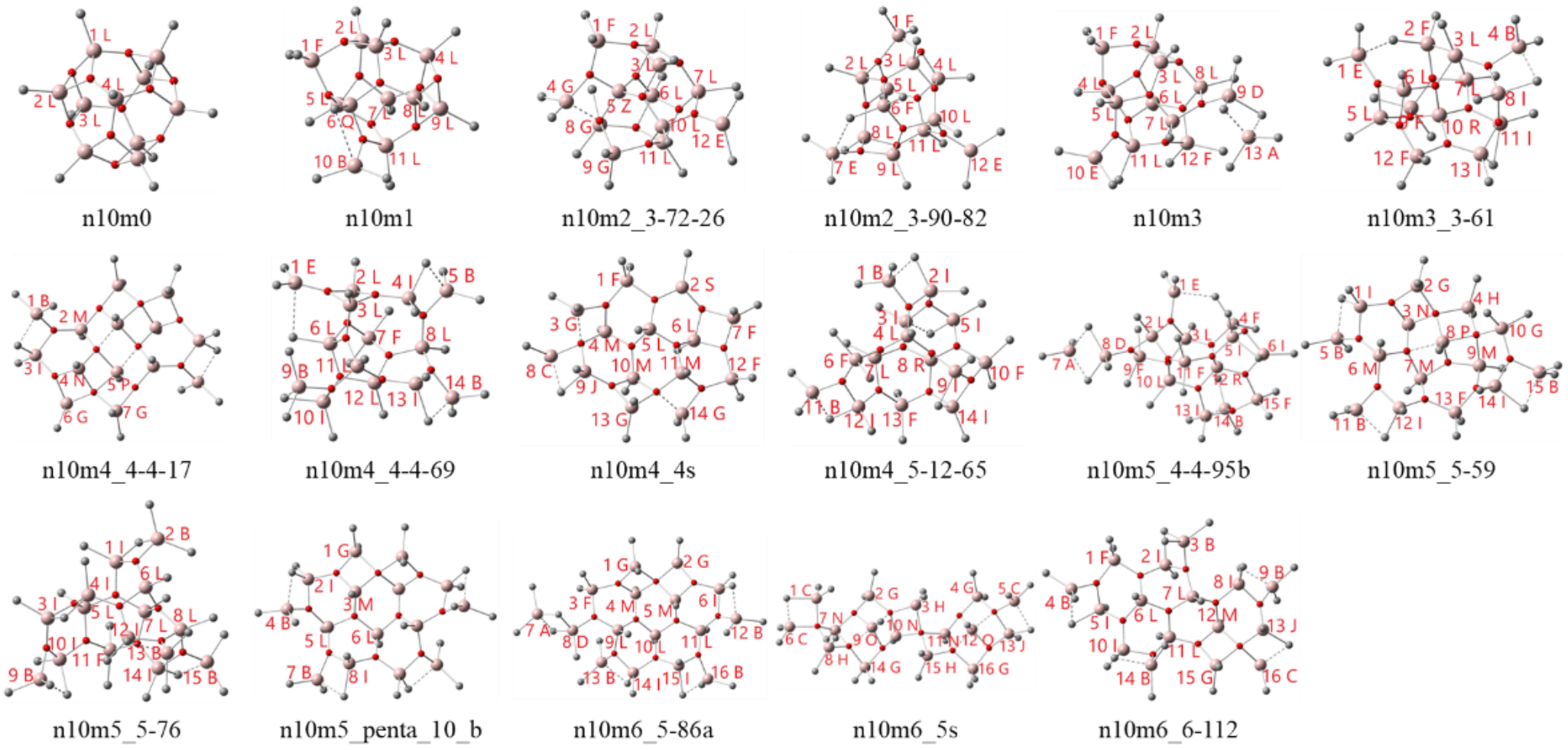

Figure S-1 – Complete MAO structures list (n = 4-10) with labeled reactive sites.

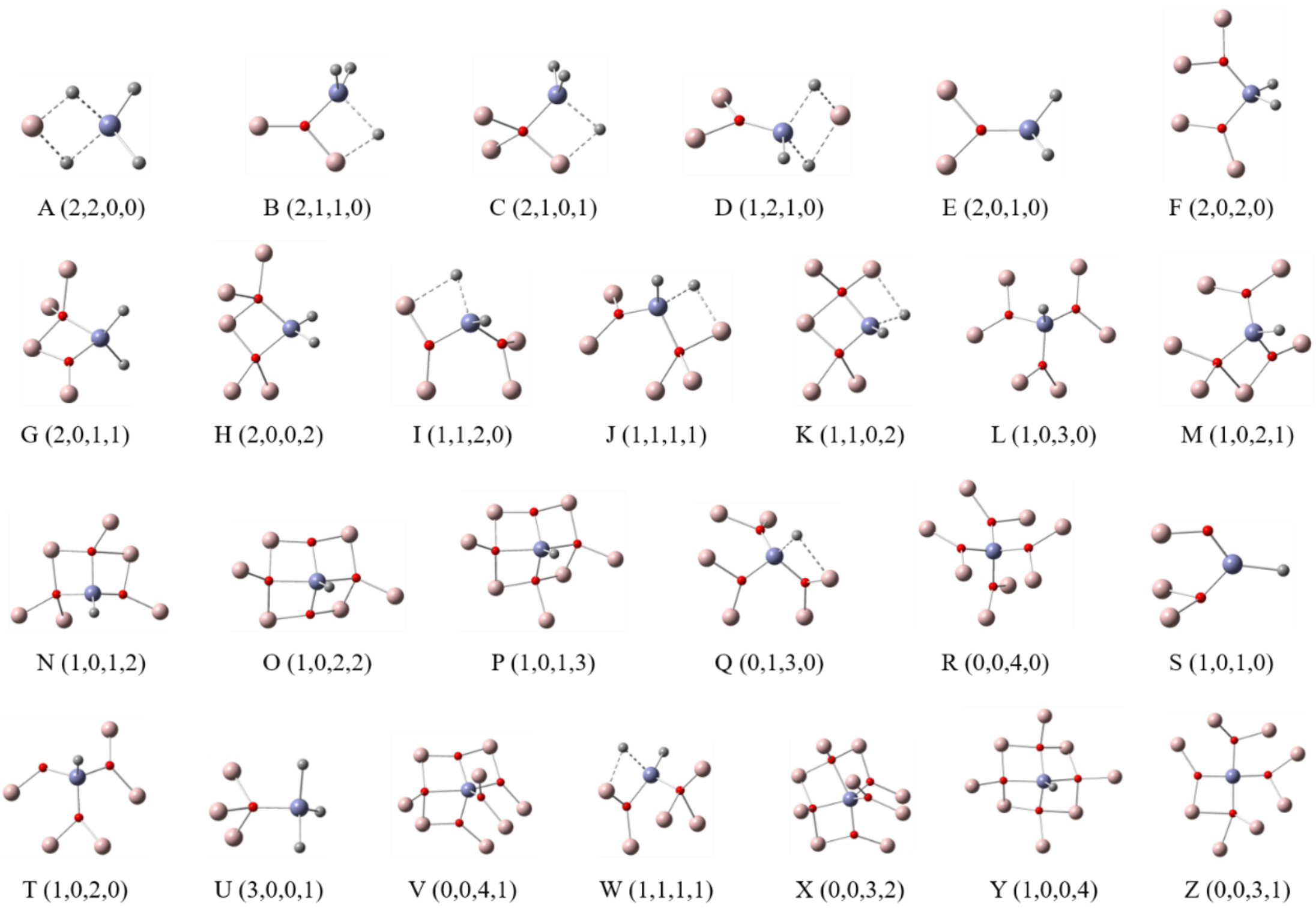

Figure S-2 – Site classifications.

In addition to the alphabetical labels, a new numerical descriptor is introduced, where the numbers in parentheses represent the counts of terminal Me groups, bridging Me groups, three-coordinate O atoms, and four-coordinate O atoms, respectively.

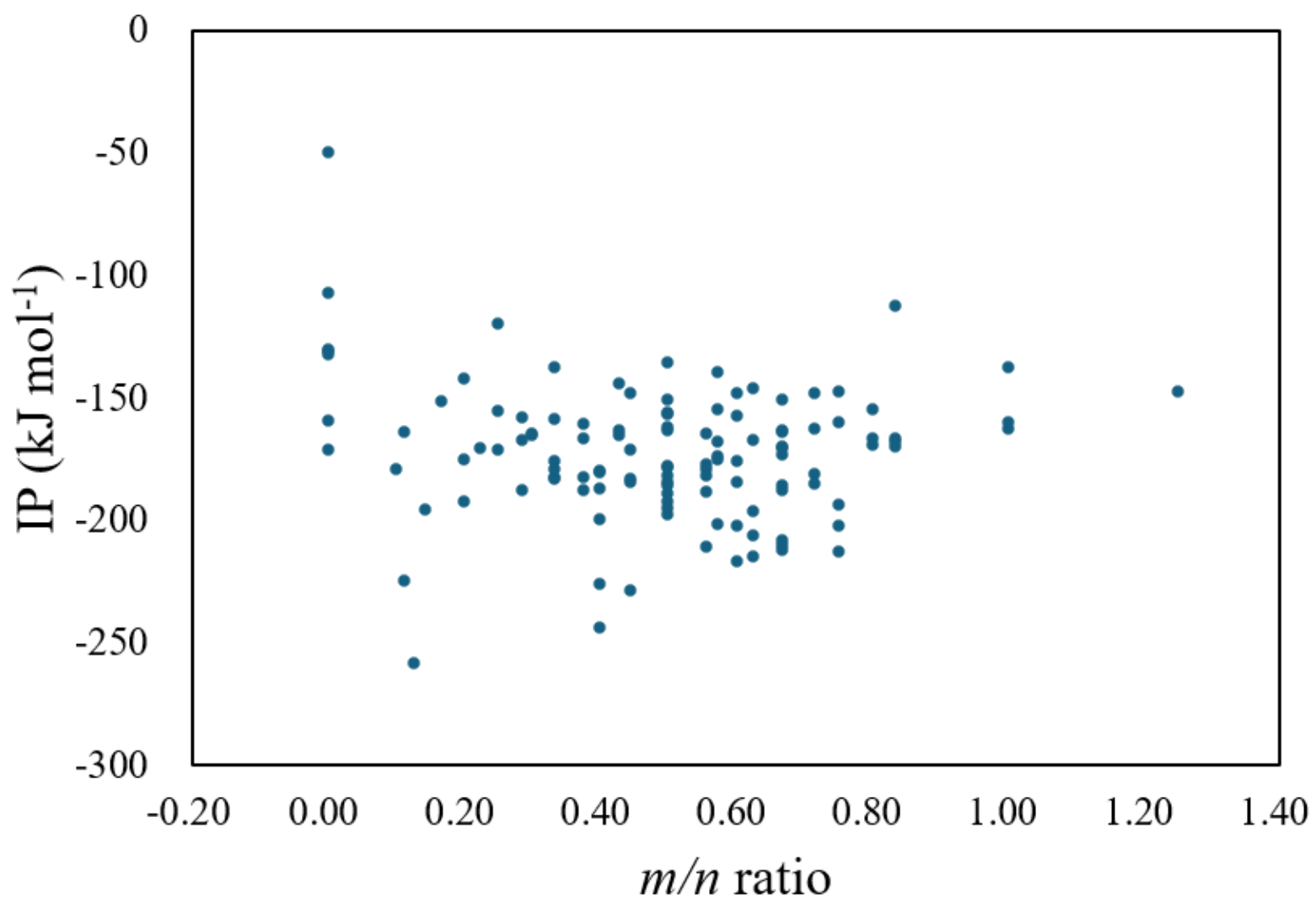

Figure S-3 – Scatter plot of the lowest IP as a function of the relative TMA content (m/n ratio).

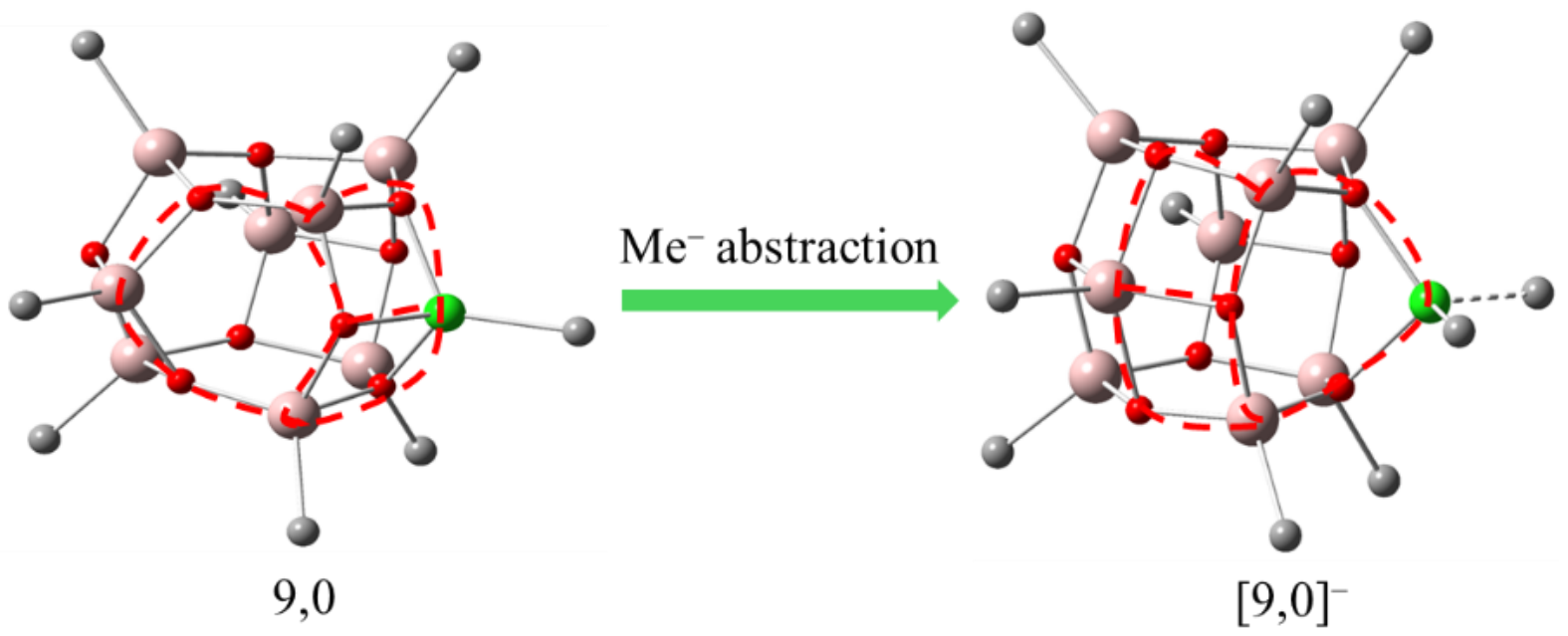

Figure S-4 – Structural comparison of 9,0 cage before and after Me$^-$ abstraction.

The fused Al_2O_2 rings were opened, and the cage structure was reorganized.

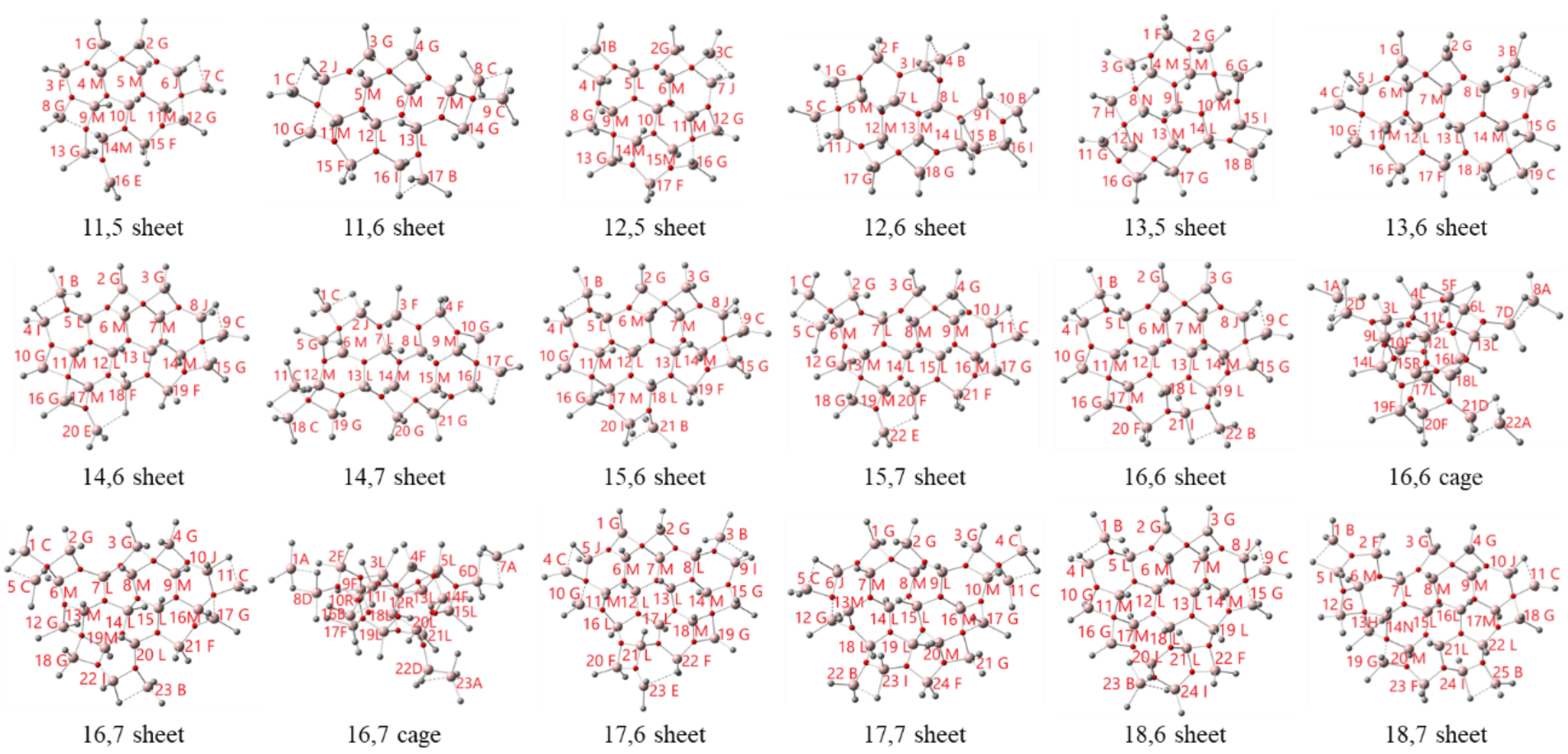

Figure S-5 – Extended MAO structures list (*n* = 11-18) with labeled reactive sites.

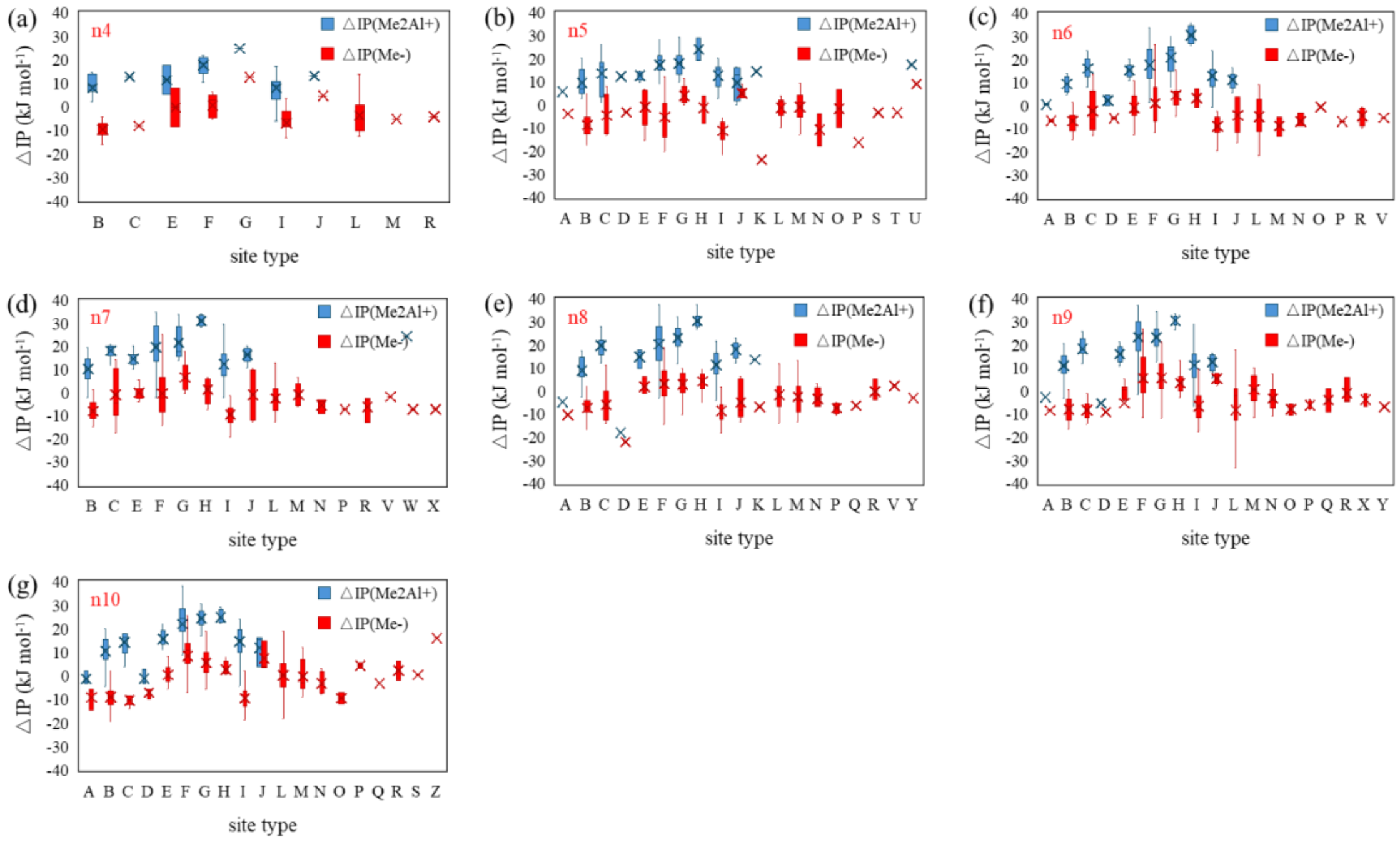

Figure S-6 – Box plots of the IP deviations of M06-2X from RI-MP2 by site classification.

The IP deviations of M06-2X/RI-MP2 for all structures with *n* = 4-10 were compared for reference. IP deviation is defined as △IP = IP (M06-2X) − IP (RI-MP2), where a positive value indicates that RI-MP2 method predicts lower ionization potentials relative to M06-2X method. As shown in Figure S-6, the ΔIPs of Me_2Al^+ cleavage at different sizes and sites are dominantly positive and generally higher than those of Me^- abstraction, indicating that RI-MP2 method provides lower ionization potential estimation under this mechanism. These systematic deviations are consistent with the findings of our recent benchmarking study [49].

Table S-1 – Gas-phase electronic energies (a.u.) at the RI-MP2/def2-TZVP level of theory for species (*n* = 4 to 10) reported in Figure 5(a). Coordinates of neutrals and anions appear in Table S-1.xyz and Table S-2.xyz, respectively.
Structures favoring Me_2Al^+ cleavage are denoted with an asterisk (*).

Neutral(*n*,*m*)	E(*n*,*m*)	[*n*,*m*]⁻/[*n*,*m*−1]⁻	Site	E([*n*,*m*]⁻)/E([*n*,*m*−1]⁻)
n4m0	-1428.330524	$[4,0]^-$	L	-1468.184484
n4m1	-1789.812813	$[4,1]^-$	L	-1829.693443
n4m1_4	-1789.805733	$[4,1]^-$	I	-1829.700011
n4m2	-2151.299391	$[4,2]^-$	I	-2191.204783
n4m2_23	-2151.297528	$[4,2]^-$	I	-2191.201930
n4m3_27	-2512.770185	$[4,3]^-$	E	-2552.661478
n4m3_161	-2512.774589	$[4,3]^-$	J	-2552.670608
n4m5_13	-3235.685777	$[4,5]^-$	B	-3275.577055

n5m0	-1785.417514	[5,0]⁻	L	-1825.313224
n5m1	-2146.951208	[5,1]⁻	F	-2186.840366
n5m2	-2508.415971	[5,2]⁻	I	-2548.322325
n5m2_22	-2508.398929	[5,2]⁻	I	-2548.320043
n5m3	-2869.883660	[5,3]⁻	M	-2909.801434
n5m3_31	-2869.888560	[5,3]⁻	I	-2909.783569
n5m3_215	-2869.884854	[5,3]⁻	E	-2909.776285
n5m4_4s	-3231.353857	[5,4]⁻	G	-3271.253510
n5m4_5s	-3231.355154	[5,4]⁻	N	-3271.253789
n5m4_40	-3231.353022	[5,4]⁻	F	-3271.247172
n5m5_2-38-54	-3592.797967	[5,5]⁻	C	-3632.694953
n5m5_4s-74a	-3592.803356	[5,5]⁻	G	-3632.699444
n5m5_6-86-33	-3592.805972	[5,5]⁻	B	-3632.693542
n6m0	-2142.580164	[6,0]⁻	L	-2182.465712
n6m1	-2504.066597	[6,1]⁻	I	-2543.959293
n6m2	-2865.539774	[6,2]⁻	B	-2905.427254
n6m2_91-53-8	-2865.537601	[6,2]⁻	I	-2905.439725
n6m2_436-12-62	-2865.544240	[6,2]⁻	I	-2905.439731
n6m3_31-41	-3227.008732	[6,3]⁻	I	-3266.901353
n6m3_76-7-8	-3227.000882	[6,3]⁻	E	-3266.895764
n6m3_91-8-62	-3226.992392	[6,3]⁻	I	-3266.900732
n6m3_436-12	-3227.003180	[6,3]⁻	I	-3266.897770
n6m4_3-30-49f	-3588.456571	[6,4]⁻	E	-3628.356278
n6m4_4-12-24a	-3588.457706	[6,4]⁻	B	-3628.355347
n6m4_4-70-52b	-3588.462530	[6,4]⁻	J	-3628.377748
n6m4_5-3-69	-3588.468174	[6,4]⁻	J	-3628.369195
n6m4_5-42-69	-3588.462811	[6,4]⁻	F	-3628.362862
n6m4_5s	-3588.468813	[6,4]⁻	J	-3628.374635
n6m5_5-3-88a	-3949.919278	[6,5]⁻	N	-3989.819042
*n6m5_5-63-69a	-3949.921903	[6,4]⁻	C	-3628.377269
n6m5_5s	-3949.927435	[6,5]⁻	N	-3989.826538
*n6m5_c	-3949.908063	[6,4]⁻	A	-3628.342785
n7m0_c3v	-2499.686594	[7,0]⁻	L	-2539.571646
n7m1	-2861.189389	[7,1]⁻	I	-2901.098971
n7m2_2-17	-3222.646908	[7,2]⁻	F	-3262.545759
n7m2_3-35-50	-3222.658214	[7,2]⁻	L	-3262.553467
n7m2_3-72-30	-3222.650465	[7,2]⁻	I	-3262.557049
n7m3_3-26-43b	-3584.125492	[7,3]⁻	I	-3624.015511
n7m3_3-62	-3584.126605	[7,3]⁻	I	-3624.024008
n7m3_3-62-48a	-3584.123703	[7,3]⁻	I	-3624.021648
n7m4_3-62c	-3945.580953	[7,4]⁻	F	-3985.479919
n7m4_4-22-49b	-3945.577637	[7,4]⁻	E	-3985.465884
n7m4_4-34-63a	-3945.583301	[7,4]⁻	M	-3985.485036
n7m4_4-67-43a	-3945.587352	[7,4]⁻	I	-3985.481304
n7m4_5-25-90	-3945.570861	[7,4]⁻	J	-3985.482865
n7m4_5-63-68	-3945.573451	[7,4]⁻	F	-3985.474668
n7m5_5-21-3a	-4307.039728	[7,5]⁻	N	-4346.945302
n7m5_5-76-3a	-4307.029830	[7,5]⁻	I	-4346.926784
n7m5_5-82	-4307.038602	[7,5]⁻	C	-4346.942594
*n7m5_5-89	-4307.038155	[7,4]⁻	B	-3985.486494

n8m0	-2856.826984	$[8,0]^-$	L	-2896.702977
n8m1_2-45-57	-3218.304757	$[8,1]^-$	L	-3258.238366
n8m2_2-61-37a	-3579.783664	$[8,2]^-$	I	-3619.683921
n8m3_3-7	-3941.243838	$[8,3]^-$	I	-3981.140158
n8m3_3-21-7	-3941.229067	$[8,3]^-$	J	-3981.135719
n8m3_3-74-54a	-3941.235169	$[8,3]^-$	B	-3981.133844
n8m3_4-91-13	-3941.228711	$[8,3]^-$	L	-3981.133196
n8m4_3-26c	-4302.700610	$[8,4]^-$	F	-4342.603668
n8m4_4-41	-4302.695176	$[8,4]^-$	M	-4342.604539
n8m4_4-63-35a	-4302.695574	$[8,4]^-$	L	-4342.592374
n8m4_4-91-24a	-4302.697262	$[8,4]^-$	I	-4342.604299
n8m4_tube	-4302.702200	$[8,4]^-$	I	-4342.588869
n8m5_4-69b	-4664.153221	$[8,5]^-$	I	-4704.052123
n8m5_4-83a	-4664.155217	$[8,5]^-$	B	-4704.045927
n8m5_5-12-53a	-4664.147407	$[8,5]^-$	J	-4704.061091
n8m5_5-68	-4664.158799	$[8,5]^-$	I	-4704.068618
n8m5_5-87	-4664.146522	$[8,5]^-$	Y	-4704.063537
*n8m6_5-104-21c	-5025.600907	$[8,5]^-$	C	-4704.066708
n8m6_5s	-5025.608905	$[8,6]^-$	N	-5065.521125
n8m6_sc	-5025.600948	$[8,6]^-$	N	-5065.517108
n9m0	-3213.952845	$[9,0]^-$	L	-3253.853147
n9m1_1-13	-3575.411154	$[9,1]^-$	M	-3615.331814
n9m1_1-54-36a	-3575.423983	$[9,1]^-$	L	-3615.321453
n9m2_2-36-55a	-3936.902257	$[9,2]^-$	L	-3976.802414
n9m3_1-20h	-4298.344922	$[9,3]^-$	L	-4338.249658
n9m3_3-25-9a	-4298.354441	$[9,3]^-$	I	-4338.259078
n9m3_3-74-39a	-4298.357581	$[9,3]^-$	I	-4338.260814
n9m4_4-68	-4659.802344	$[9,4]^-$	G	-4699.724590
n9m4_4-93-65a	-4659.806166	$[9,4]^-$	E	-4699.711413
n9m4_4-97-83a	-4659.816096	$[9,4]^-$	I	-4699.720915
n9m4_4s	-4659.805739	$[9,4]^-$	B	-4699.705980
n9m4_tube	-4659.807495	$[9,4]^-$	I	-4699.698993
n9m5_5-5-3	-5021.272417	$[9,5]^-$	G	-5061.175870
n9m5_5-13	-5021.266683	$[9,5]^-$	N	-5061.173575
n9m5_5-54	-5021.266320	$[9,5]^-$	I	-5061.164058
n9m5_5-93	-5021.267191	$[9,5]^-$	L	-5061.171436
n9m5_5s	-5021.260880	$[9,5]^-$	N	-5061.163478
n9m5_5-sc-106	-5021.259857	$[9,5]^-$	J	-5061.175315
n9m6_5-5-100a	-5382.728297	$[9,6]^-$	G	-5422.625634
n9m6_5-44a	-5382.722539	$[9,6]^-$	M	-5422.614943
n9m6_5s	-5382.718306	$[9,6]^-$	Y	-5422.632598
n9m6_6-110	-5382.713130	$[9,6]^-$	G	-5422.619825
n9m6_sc	-5382.721931	$[9,6]^-$	N	-5422.637879
n10m0	-3571.060638	$[10,0]^-$	L	-3610.945439
n10m1	-3932.545730	$[10,1]^-$	B	-3972.449120
n10m2_3-72-26	-4294.000607	$[10,2]^-$	L	-4333.908850
n10m2_3-90-82	-4294.015170	$[10,2]^-$	L	-4333.916996
n10m3	-4655.473868	$[10,3]^-$	L	-4695.372036
n10m3_3-61	-4655.472591	$[10,3]^-$	L	-4695.370374
n10m4_4-4-17	-5016.923546	$[10,4]^-$	M	-5056.834766

n10m4_4-4-69	-5016.923617	[10,4]⁻	E	-5056.827498
n10m4_4s	-5016.911770	[10,4]⁻	S	-5056.839703
n10m4_5-12-65	-5016.940877	[10,4]⁻	I	-5056.844542
n10m5_4-4-95b	-5378.385259	[10,5]⁻	I	-5418.282631
n10m5_5-59	-5378.382626	[10,5]⁻	I	-5418.293041
n10m5_5-76	-5378.386364	[10,5]⁻	I	-5418.292157
n10m5_penta_10_b	-5378.391709	[10,5]⁻	I	-5418.294582
*n10m6_5-86a	-5739.845347	[10,5]⁻	A	-5418.307675
n10m6_5s	-5739.834064	[10,6]⁻	N	-5779.736136
n10m6_6-112	-5739.839181	[10,6]⁻	G	-5779.751258

Table S-2 – Gas-phase electronic energies (a.u.) at the RI-MP2/def2-TZVP level of theory for species reported in Table 1. Coordinates of neutrals and anions appear in Table S-3.xyz

Neutral(*n*,*m*)	E(*n*,*m*)	Site type	E([*n*,*m*−1]⁻)	Site type	E([*n*,*m*]⁻)
11,5 4-C sheet	-5735.498562	C	-5413.953168	G	-5775.410914
11,6 4-C sheet	-6096.961474	B	-5775.408098	M	-6136.863383
12,5 4-C sheet	-6092.626382	C	-5771.079348	G	-6132.534487
12,6 4-C sheet	-6454.078013	C	-6132.525861	B	-6493.977058
13,5 4-C sheet	-6449.730314	H	-6128.180606	G	-6489.645326
13,6 4-C sheet	-6811.201510	C	-6489.653358	G	-6851.107790
14,6 4-C sheet	-7168.316614	C	-6846.762489	G	-7208.221797
14,7 4-C sheet	-7529.769949	C	-7208.222994	—	—
15,6 4-C sheet	-7525.438773	C	-7203.888571	G	-7565.343055
15,7 4-C sheet	-7886.886721	C	-7565.333125	—	—
16,6 4-C sheet	-7882.563790	C	-7561.018562	G	-7922.474835
16,6 cage	-7882.556869	A	-7561.009053	F	-7922.414040
16,7 4-C sheet	-8244.009364	C	-7922.462295	G	-8283.916208
16,7 cage	-8244.006393	A	-7922.455166	B	-8283.887347
17,6 4-C sheet	-8239.670231	C	-7918.124905	G	-8279.581417
17,7 4-C sheet	-8601.132523	C	-8279.587587	G	-8641.043742
18,6 4-C sheet	-8596.792642	C	-8275.247075	G	-8636.703787
18,7 4-C sheet	-8958.240586	C	-8636.696065	G	-8998.152185
